# A subcellular biochemical model for T6SS dynamics reveals winning competitive strategies

**DOI:** 10.1093/pnasnexus/pgad195

**Published:** 2023-06-13

**Authors:** Yuexia Luna Lin, Stephanie N Smith, Eva Kanso, Alecia N Septer, Chris H Rycroft

**Affiliations:** Flexible Structures Laboratory, Ecole Polytechnique Fédérale de Lausanne (EPFL), Rte Cantonale, Lausanne CH-1015, Switzerland; John A. Paulson School of Engineering and Applied Sciences, Harvard University, 29 Oxford Street, Cambridge, MA 02138, USA; Department of Earth, Marine, and Environmental Sciences, University of North Carolina, 121 South Road, Chapel Hill, NC 27599, USA; Department of Aerospace and Mechanical Engineering, University of Southern California, 3650 McClintock Ave, Los Angeles, CA 90089, USA; Department of Earth, Marine, and Environmental Sciences, University of North Carolina, 121 South Road, Chapel Hill, NC 27599, USA; John A. Paulson School of Engineering and Applied Sciences, Harvard University, 29 Oxford Street, Cambridge, MA 02138, USA; Department of Mathematics, University of Wisconsin–Madison, 480 Lincoln Drive, Madison, WI 53706, USA; Computational Research Division, Lawrence Berkeley Laboratory, 1 Cyclotron Rd, Berkeley, CA 94720, USA

**Keywords:** *Aliivibrio fischeri* type VI secretion system, competition, viscosity activation, sheath number distribution, agent-based model

## Abstract

The type VI secretion system (T6SS) is a broadly distributed interbacterial weapon that can be used to eliminate competing bacterial populations. Although unarmed target populations are typically used to study T6SS function in vitro, bacteria most likely encounter other T6SS-armed competitors in nature. However, the connection between subcellular details of the T6SS and the outcomes of such mutually lethal battles is not well understood. Here, we incorporate biological data derived from natural competitors of *Vibrio fischeri* light organ symbionts to build a biochemical model for T6SS at the single-cell level, which we then integrate into an agent-based model (ABM). Using the ABM, we isolate and experiment with strain-specific physiological differences between competitors in ways not possible with biological samples to identify winning strategies for T6SS-armed populations. Through in vitro experiments, we discover that strain-specific differences exist in T6SS activation speed. ABM simulations corroborate that faster activation is dominant in determining survival during competition. Once competitors are fully activated, the energy required for T6SS creates a tipping point where increased weapon building and firing becomes too costly to be advantageous. Through ABM simulations, we identify the threshold where this transition occurs in the T6SS parameter space. We also find that competitive outcomes depend on the geometry of the battlefield: unarmed target cells survive at the edges of a range expansion where unlimited territory can be claimed. Alternatively, competitions within a confined space, much like the light organ crypts where natural *V. fischeri* compete, result in the rapid elimination of the unarmed population.

**Significance Statement**

All life forms, including bacteria, fight for limited space and resources. Although interbacterial battles occur at microscopic scales, these competitions within host colonization sites may have far-reaching effects on host health. Yet little is known about how some bacteria strategically use their molecular weapons to dominate over a competitor with a well-matched arsenal. To fill this knowledge gap, we used biological data to build a multiscale computational model that can simulate the lethal battles observed in natural bacterial competitors. We used this model to explore how the following factors balance against one another to determine the winner: the speed to activate building of weapons, the speed to build and fire weapons, and the cost of building these weapons.

All life forms compete with one another in what Charles Darwin referred to as “the struggle for life” (1). Indeed, fierce battles for limited space and resources can be observed across biological complexity, from single-cell organisms to humans. These battles often determine which population will proliferate and which will be excluded. Therefore, organisms of all size scales have evolved diverse strategies to wage war on their rivals and increase the probability of their success.

Microbial genomes encode an incredible arsenal of interbacterial weaponry (2). Some, e.g. the broadly distributed type VI secretion system (T6SS), have been shown to be useful in competing for colonization sites within a host niche (3–11). T6SSs resemble a molecular syringe and are thought to have evolved from bacteriophage contractile tails (12, 13). T6SS-containing cells build a sheath-and-tube structure that extends the width of a cell and is anchored into the cell wall and membrane (14). When a T6SS${}^{+}$ cell comes in contact with a target cell, the sheath is contracted, the inner tube and toxins are propelled into the neighboring cell, resulting in death and lysis of the competitor (15–19). Recent work has revealed that the T6SS arsenal also functions in contact-independent ways (20). Although genome sequencing allows us to identify the bacterial strains that harbor T6SS interbacterial weapons (21), we are unable to predict which microbial population will dominate in an ecologically relevant battle based on genetic data alone (22). To do so requires a more detailed and holistic understanding of the T6SS function, dynamics, and regulation and how these factors, combined with environmental ones, influence competitive outcomes. The goal of this work is to gain insight into how differences in T6SS function at the individual cell level can influence the survival of that genotype at the population level when facing another T6SS-armed competitor.

Numerous works contribute to uncovering the diversity in the regulation of T6SS expression, as well as its function and behavior in various bacteria (15, 20, 23–37). Other studies investigate the effect of T6SS-mediated competition on spatial organization and population dynamics in vivo and in vitro (8, 28, 38–41). In natural niches like the light organ of the Hawaiian bobtail squid, *Euprymna scolopes*, T6SS${}^{+}$*Vibrio fischeri* has been shown to eliminate unarmed competitors and singularly colonize entire crypt spaces (8, 11, 19, 28, 38, 42, 43). In vitro, studies of T6SS-dependent competition find that mutually armed strain pairings often result in coexistence through the formation of coarse, spatially separated microcolonies in which T6SS attacks occur on the borders between strains (8, 39, 40).

Indeed, recent works have shown how T6SS function can be influenced by several biochemical factors. For example, studies have shown that the availability of T6SS structure proteins affects the number, form, and function of the T6SS apparatus (29, 30, 44), and the speed of cell lysis induced by T6SS effectors can fine-tune the effectiveness of the T6SS apparatus as a lethal weapon (17, 26, 45). Many of these works combine experiments with computational approaches, such as agent-based modeling, to study such complex living systems. Agent-based models (ABMs) have been widely applied to studying population dynamics and spatial organization in a broad range of contexts (46–53). When applied to modeling T6SS-dependent competitions between bacterial populations (17, 26, 27, 39, 54–56), ABMs have yielded insightful results regarding the survival of target populations under T6SS attack (17, 54) and the evolution of T6SS attack strategies such as tit-for-tat (27, 56).

Existing T6SS ABMs have established well-accepted interaction rules among T6SS-dueling cells (17, 26, 27, 39, 40, 54, 56). For example, T6SS weapons are fired at a given rate, and they hit a target cell with some probability; after receiving a certain number of attacks, the target cell dies and subsequently lyses. These interactions are often modeled as probabilistic events at the cellular level. For example, to model firing, a random number is drawn from a Poisson distribution (17, 56) or a uniform distribution (39) to represent the number of firing events within a time interval without strong experimental evidence supporting these distributions. Bacteria are also known to regulate T6SS activity according to environmental stimuli (34, 41–43, 57–59). However, this aspect has largely been neglected in modeling. Together, this underscores the lack of a unified mathematical framework to model how T6SS structure numbers and firing frequencies are regulated that is also based on experimental data.

Here, we use *V. fischeri* as a model organism to fill this knowledge gap and connect physiologically relevant T6SS biochemical factors to competitive outcomes (Fig. 1A). Our study builds on previous findings showing that when two T6SS-armed *V. fischeri* strains compete, one is eventually eliminated, albeit not as quickly as an unarmed population (8). These findings revealed that (1) strain-specific differences exist among T6SS${}^{+}$ populations and affect competitive outcomes and (2) *V. fischeri* serves as a tractable model system to study how strain-level diversity influences T6SS-mediated killing between natural competitors. In this work, we performed assay experiments on *V. fischeri* light organ isolates from *E. scolopes*, which are natural competitors of the squid light organ and conditionally express a T6SS encoded on chromosome II (T6SS2) (8, 11, 28, 60, 61). Upon entering a high viscosity or surface-associated environment, *V. fischeri* can activate and engage T6SS2 to kill under laboratory conditions (41–43). Because the T6SS2 of *V. fischeri* can be controlled by culture conditions, we were able to quantify the strain-specific differences in speed of T6SS activation, number of sheaths per cell, and killing rates.

**Fig. 1.**
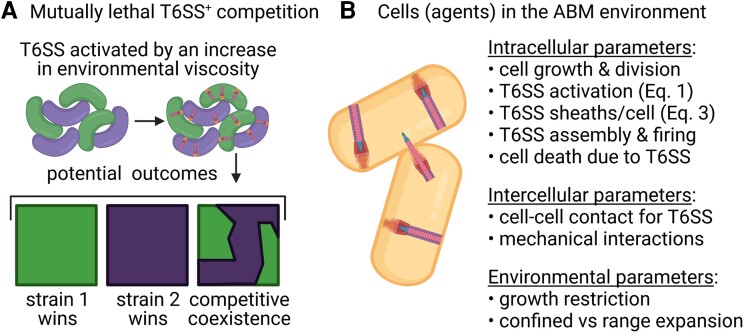
Schematics of experimental and computational systems used in this study. A) A schematic of a coincubation between two mutually lethal T6SS${}^{+}$ strains of *V. fischeri* on agarose pads. Cells raised in liquid culture first undergo surface activation after plating. The competitive outcome depends on intraspecific physiological variations, such as activation rate and the speed at which cells build and fire T6SS apparatuses. B) A schematic of a computational cell in the in-house ABM environment. Besides cellular growth and division, a cell (or an agent) also undergoes internal reactions governed by the subcellular T6SS model, subject to environmental factors such as cell-cell contact, spatial confinement, and growth restriction due to the availability of space. For more details of the ABM, see SI Appendix Section 3 and 4. This figure is created with Biorender.com.

Although any of these factors could tip the balance in favor of one strain in competition, we are unable to manipulate each factor, alone or in combination, in biological experiments. Therefore, we leverage the detailed microscopy data to build a model of subcellular T6SS dynamics and map experimental measurements to model parameters. We then integrate this subcellular model in an off-lattice ABM (Fig. 1B) and validate the ABM against experimental data. An ABM is particularly suitable for investigating the connection between the subcellular dynamics of T6SS and the competitive strength of a T6SS${}^{+}$ population because it bridges the scale of individual cells and that of the population as a whole. The choice of using an ABM also helps define the scale at which we approach modeling of the subcellular dynamics. We restrict our model to the scale of individual T6SS structures, which has the dual benefits of being easily tracked in individual cells in the ABM, and easily visualized in vitro using live-cell fluorescence microscopy. By systematically varying the T6SS parameters in the ABM, we investigate how competitive outcomes depend on T6SS activation level, the number of T6SS structures cells harbor, how fast structures can be built and utilized, the cost of building structures, as well as the spatial geometry of the competition arena.

## Results

### Competition outcomes differ due to intraspecific variations in T6SS killing dynamics

The ability of lethal strains of *V. fischeri*, which encode a strain-specific T6SS genomic island (T6SS2), to produce T6SS structures and kill target cells is dependent on environmental stimuli (41–43, 61). In liquid culture, *V. fischeri* T6SS is functionally inactive, while the exposure to a high viscosity medium such as hydrogel or an agar surface causes the cells to activate T6SS protein expression and structure formation (41). We hypothesized that the response time to surface activation might differ among lethal strains of *V. fischeri* and that these variations may affect competition outcomes between two lethal strains (Fig. 1A). To begin testing this hypothesis, we coincubated the T6SS2-encoding *V. fischeri* strains ES401 and FQ-A002 (8) on LBS agar plates following two different preparations: clonal cultures of each strain were first incubated for 6 h either in (1) LBS liquid medium where T6SS2 activity is low or (2) on LBS agar plates where T6SS2 activity is increased. Treatments where strains were incubated in liquid are referred to as “unprimed” because both strains come from a “T6SS off” condition and must activate T6SS at the start of their coincubation on agar surfaces, whereas strains incubated on agar are referred to as “primed” because these strains have fully activated T6SSs when they begin their coincubation on agar surfaces. We predicted that if the response time to surface activation is different between these two strains, then the strain that can more quickly activate T6SS to begin killing its competitor will dominate in the unprimed condition.

**Fig. 2.**
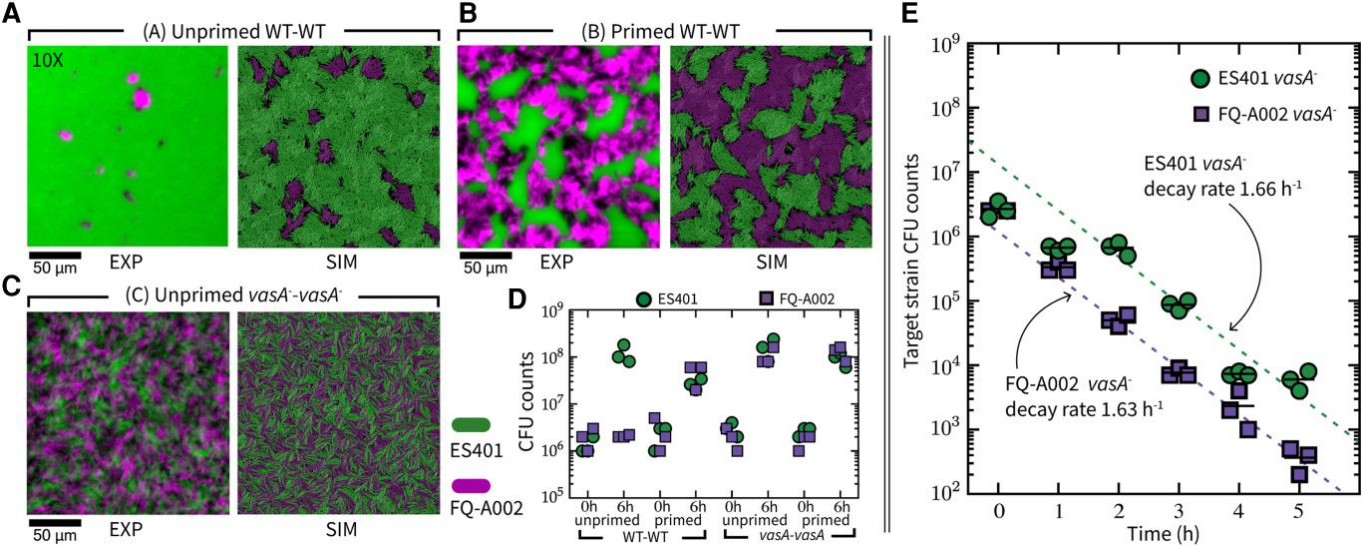
Competition outcomes vary due to intraspecific variations in T6SS killing dynamics. A–C) Fluorescence microscopy images of ES401 and FQ-A002 strains at 24 h following coincubation on LBS agar, compared side by side with representative ABM simulation images. The ES401 strain harbored the GFP-encoding plasmid pVSV102 (green), and the FQ-A002 strain harbored the dsRed-encoding plasmid pVSV208 (magenta). Microscopy images were taken at 10X; scale bars are 50 µm for all microscopy and simulation images. A) Wildtype (WT) vs. wildtype pair under unprimed treatment. In the microscopy images, ES401 occupied 99.3% of the total area and FQ-A002 0.7%. In simulations, ES401 occupies 91.4%, and FQ-A002 1.8%. B) WT vs. WT pair under primed treatment. In the microscopy images, ES401 occupied 46.6% of the total area and FQ-A002 53.4%. In simulations, ES401 occupies 53.2%, and FQ-A002 46.8%. C) *vasA*${}^{-}$ vs. *vasA*${}^{-}$ pair under unprimed treatment. In microscopy images, ES401 occupied 48.7% of the total area and FQ-A002 51.3%. In simulations, ES401 occupies 51.2%, and FQ-A002 48.8%. The *vasA*${}^{-}$ vs. *vasA*${}^{-}$ pair under primed treatment gives a similar outcome to C) experimentally; in simulation, it is identical to *vasA*${}^{-}$ vs. *vasA*${}^{-}$ under unprimed treatment, hence not repeated. All experiments were performed three times; all simulations were repeated 100 times. For additional experiment and simulation data, see SI Appendix Section 1, Fig. S1, movies S1 and S2. For simulation parameters, see SI Appendix Tables S1 and S2. D) Total CFU counts of each strain in each coincubation at 0 h and 6 h following coincubation on LBS agar. E) Total target strain CFU counts taken each hour for 5 h in unprimed coincubation between either wildtype FQ-A002 and ES401 *vasA*${}^{-}$ or wildtype ES401 and an FQ-A002 *vasA*${}^{-}$ . CFU data taken after time T≥2h of either strain is fit to an exponential decay model aexp(−bt) with a,b as fitting parameters. Data points at 0 h and 1 h are excluded. Decay rate *b* is reported for both strains.

When unprimed wildtype ES401 and FQ-A002 were coincubated on LBS agar plates, microscopy images and colony forming unit (CFU) counts revealed that ES401 outcompeted FQ-A002 at the population level and only small microcolonies of FQ-A002 remained after 24 h of coincubation (Fig. 2A and D). Percentages of occupied area are calculated using the microscopy images; ES401 occupies 99.3% of the total area, compared with only 0.7% occupied by FQ-A002. However, when primed wildtype ES401 and FQ-A002 were coincubated, the two strains coexisted with comparable CFU counts and formed coarse, spatially separated microcolonies (Fig. 2B and D). The percentages of occupied area are 46.6% and 53.4%, for ES401 and FQ-A002, respectively. To determine whether this effect was dependent on T6SS2 activity, we used ES401 and FQ-A002 strains with a disruption in the *vasA*_*2* gene, which encodes a baseplate protein in the T6SS2 complex and is required for T6SS-dependent killing (8, 41). These *vasA* mutant (*vasA*${}^{-}$) strains were then coincubated in both primed and unprimed conditions. Regardless of coincubation conditions, both *vasA*${}^{-}$ strains coexisted and were well-mixed, with no spatial separation between strains (Fig. 2C and D). The percentages of the occupied area from each *vasA*${}^{-}$ strain in both conditions are 48.7% and 51.3%, for ES401 *vasA*${}^{-}$ and FQ-A002 *vasA*${}^{-}$, respectively. These findings reveal that one lethal strain (ES401) can dominate over another lethal strain (FQ-A002) when both competitors have inactive T6SSs at the onset of competition, and the results support our hypothesis that surface activation timing affects competitive outcomes, which warrant further investigation.

Based on the above findings, we reasoned that ES401 might activate its T6SS and begin killing target cells before FQ-A002, thus providing it an advantage in competition. To isolate the killing dynamics of both strains under the unprimed condition and to see whether the difference in killing rates after T6SS activation can be driving the observed dominance of ES401, we directly quantified their ability to eliminate a nonlethal target population over time. Specifically, we hypothesized that an unarmed target population could survive longer and in larger numbers when coincubated with a slower activating lethal strain. To test this hypothesis, we competed either (1) wildtype FQ-A002 vs. ES401 *vasA*${}^{-}$ , or (2) wildtype ES401 vs. FQ-A002 *vasA*${}^{-}$ strains by spotting unprimed mixtures of each treatment on LBS agar plates and obtaining CFU counts of the lethal (wildtype) and the unarmed target (*vasA*${}^{-}$) strains every hour during a 5 h coincubation period. When the target strain CFUs were plotted over time for each coincubation, we observed that there was an approximately 10-fold drop in target CFUs between 1 h and 2 h in the coincubation between wildtype ES401 vs. FQ-A002 *vasA*${}^{-}$ target, whereas the ES401 *vasA*${}^{-}$ target CFUs were maintained between 1 h and 2 h in the coincubation with the wildtype FQ-A002 (Fig. 2E), suggesting that ES401 begins killing target before FQ-A002. From 2 h onward, we observed that the target populations in both treatments declined exponentially at approximately the same rate, i.e. ∼exp(−(1.6 h^−1^)*t*), where *t*∈[2 h, 5 h] is time after spotting (Fig. 2E), suggesting both of the wildtype ES401 and FQ-A002 lethal strains kill at comparable rates after T6SS activation. When the CFUs of wildtype ES401 and wildtype FQ-A002 were plotted, we found that both strains exhibited an initial decline during 0 h to 1 h, similar to what was observed for the *vasA*${}^{-}$ target strains, suggesting this initial drop in CFU counts is independent of T6SS killing, and perhaps due to transition from liquid to surface growth (Fig. 2E, SI Appendix Fig. S2). Moreover, the wild-type ES401 and FQ-A002 strains had similar growth rates under these conditions (SI Appendix Fig. S2); thus, a difference in growth rate does not account for the differences in the target populations between treatments.

Taken together, these results suggest that wildtype ES401 is more effective in eliminating nonlethal targets when unprimed than wildtype FQ-A002. Combined with our previous observation that wildtype ES401 outcompetes wildtype FQ-A002 in an unprimed competition, these results support our hypothesis that variations in competitive outcomes are driven by a strain-specific T6SS activation response to surfaces. To investigate this surface response in *V. fischeri*, we performed further experiments to quantify the percentage of cells in a population with T6SSs and the number of structures per cell over time.

### Quantifying strain-specific differences in T6SS activation dynamics

**Fig. 3.**
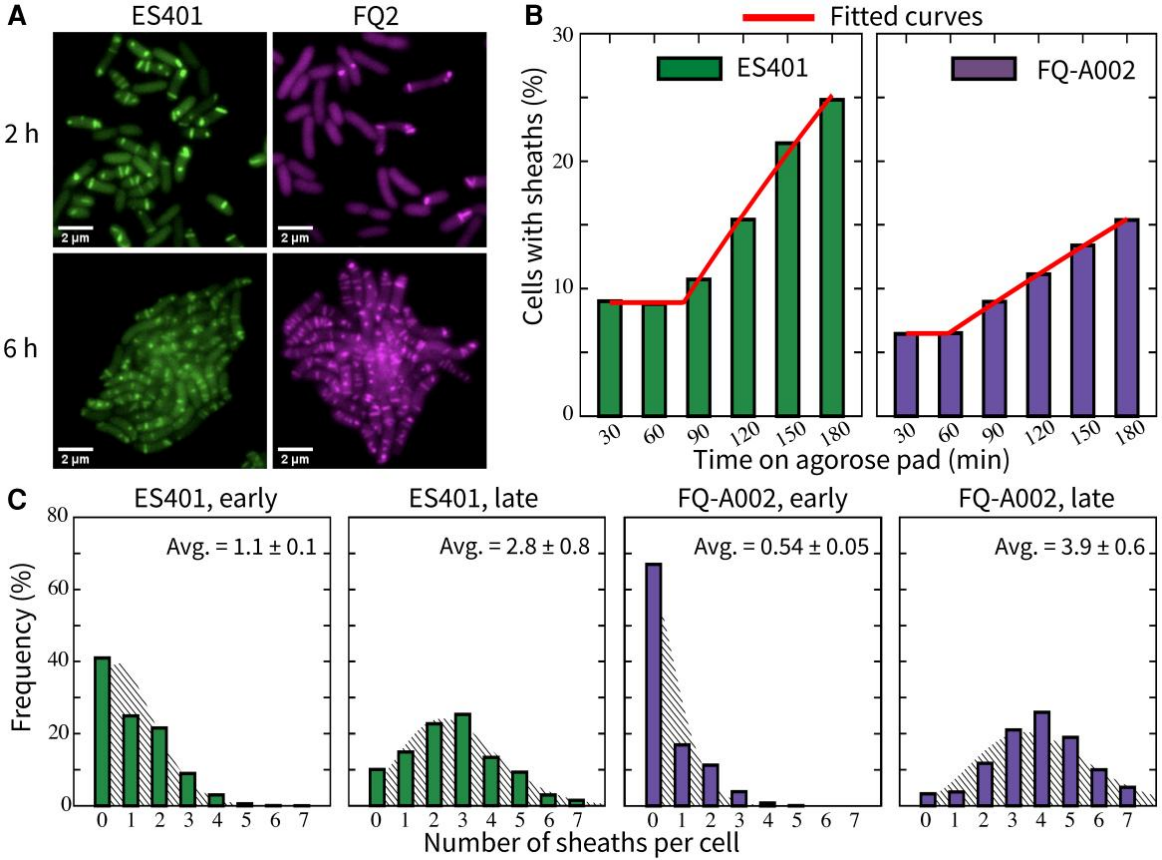
*Vibrio fischeri* exhibits strain-specific T6SS dynamics over time during surface activation. A) Representative fluorescence microscopy images of ES401 (left panels) and FQ-A002 (right panels) cells harboring VipA-GFP expression vector after incubation on an agarose pad for either 2 h or 6 h. B) Percentage of ES401 or FQ-A002 cells that contain at least one sheath at 30 min intervals for 3 h. A minimum of 680 total cells were analyzed for each treatment across five fields of view for two biological replicates. All combined data is shown. Parameters in Eq. (2) are estimated to be $\left(p_{0},\tau_{+},\lambda_{+})=(10\%,1.34\;h,0.118\;h^{-1}\right)$ for ES401, and $\left(p_{0},\tau_{+},\lambda_{+})=(5\%,1\;h,0.05\;h^{-1}\right)$ for FQ-A002. C) Distribution of the number of VipA-GFP sheaths per cell in either ES401 or FQ-A002 at early (2 h) or late (6 h) stages of incubation on an agarose pad. These data are overlaid with a Poisson distribution with the corresponding mean. Chi-squared tests comparing the experimental distribution and the Poisson distribution yield, from left to right, P<.001,P=.271,P<.001,P<.001. Chi-squared test comparing the two 6 h distributions yield P<.001. At least 2700 cells are analyzed for sheath distributions at 2 h, and at least 260 cells are analyzed at 6 h. All agarose pads were made by supplementing liquid LBS with 2% agarose and 0.5 mM IPTG (isopropyl-β-D-thiogalactopyranoside). A and C) show data from the same experiments, repeated twice, and all combined data are shown, B) shows data from a separate experiment; for details, see **Materials and methods**.

In a T6SS complex, VipAB/TssBC multimers comprise the outer component of the sheath structure; thus, VipA has been used in multiple systems as a target for visualization of sheath dynamics through the use of a fluorescently-tagged VipA/TssB fusion constructs (16, 29, 62), including an IPTG-inducible VipA-GFP expression vector in *V. fischeri* (8, 23). To visualize T6SS activation dynamics, we grew overnight cultures of wildtype ES401 or FQ-A002 strains harboring the VipA-GFP expression vector to an OD${}_{600}∼1.5$ and spotted them onto an agarose pad supplemented with 0.5 mM IPTG immediately prior to imaging. We then took green fluorescence images of VipA-GFP expressed in either ES401 or FQ-A002 for our analyses (Fig. 3A). Note that the color of each strain is due to postprocessing in the microscopy images for visualization. We categorized a cell as “T6SS activated” when we observed at least one sheath within the cell. To quantify the rate of T6SS activation for each strain, we measured the proportion of activated cells in the green fluorescence images taken at regular intervals between 0.5 and 3 h after initial spotting (Fig. 3B). These measurements revealed that the proportion of activated cells in both ES401 and FQ-A002 remained stable at low levels for approximately 1 h after plating. Directly from the liquid cultures, approximately 10% of ES401 cells and 5% of FQ-A002 cells have visible T6SS sheaths. After this initial waiting period, we observed the activated proportion increased over time in both ES401 and FQ-A002, but the rate of increase was approximately twice as high in ES401 compared with FQ-A002, suggesting ES401 activates T6SS structure assembly more quickly than FQ-A002.

We reasoned that the number of T6SS structures in the T6SS arsenal of a strain might also impact competition outcomes in either positive or negative ways, as more T6SS structures might allow a strain to kill faster, but this advantage could come at an energetic cost. Therefore, we next quantified the number of sheaths per cell over time after *V. fischeri* cells are spotted on agar surfaces. We incubated ES401 and FQ-A002 clonally on an agarose surface, as described above. For each strain, we counted the number of sheaths per cell for a given population in the green fluorescence images at an early stage after plating (2 h) and at a late stage (6 h) and plotted the frequency of having 0–7 sheaths at each time point (Fig. 3C). For both strains, the average number of sheaths per cell increased between 2 and 6 h on surfaces: ES401 went from an average of 1.1 sheaths per cell at 2 h to 2.8 sheaths per cell at 6 h, and FQ-A002 went from 0.5 at 2 h to 3.9 sheaths per cell at 6 h.

The sheath distributions of ES401 and FQ-A002 at 6 h are shown to be statistically different (Fig. 3C). We found that the experimental sheath distribution of ES401 at 6 h is similar to a Poisson distribution, which has been used in previous computational models of T6SS (17, 56) to represent the number of firing events. However, each of the other experimental sheath distributions in Fig. 3C is statistically different from the Poisson distribution with the corresponding mean.

Taken together, these results suggest that the subcellular T6SS dynamics in *V. fischeri*, including baseline activation level in liquid, the rate of surface activation, and likely the number of sheaths per cell, exhibit strain-specific variations. Moreover, a Poisson distribution is not sufficient to represent the sheath distribution in all strains and all activation states. The reasons may be attributed to the complex couplings between activation, assembly, and firing, as well as the cell cycle and other regulatory factors. We turn to mathematical and computational modeling to systematically understand the effects of the strain-specific variations in T6SS-related factors on the outcomes of intraspecific competition.

### A two-stage subcellular biochemical model of T6SS dynamics

We develop a biochemical model of T6SS, which consists of stochastic processes in two stages: (1) activation and (2) structure assembly and deployment. This type of multi-stage model based on stochastic processes is an established approach for modeling transcription and translation (63–66), and eukaryotic organelle synthesis (67), but it has not been applied to subcellular T6SS structures.

#### Activation stage

We define activation as the cell being in a state ready to assemble T6SS structures. We denote the state of T6SS activation with G, such that $G^{-}$ and $G^{+}$ indicate the inactive and the active states, respectively. A cell in the inactive state $G^{-}$ can be switched on at a constant rate $\lambda_{+}$,


(1) $$G^{-}\overset{\lambda_{+}}{⟶}G^{+}.$$

Here, we have abstracted into a single stochastic process the myriad processes that induce a T6SS-activated state in a cell, e.g. sensing environmental signals which, depending on the system, could include certain chemical species, temperature, colonization of macrophages or viscosity (34, 41, 57–59), as well as translation and accumulation of T6SS proteins (29, 30). We have also neglected the effect of the cell cycle on DNA copy number, cell volume, or chemical concentration (63–65). Cell cycle also affects the activation state of the cells and T6SS structure numbers per cell. Although we did not directly test whether daughter cells inherit their mother’s activation state, our results in Fig. 3 suggest this is likely the case as only a small percentage of cells (5–10%) at 6 h have no observed sheaths. In addition, division reduces the number of structures in the daughter cells, changing the structure number distribution in a population. To circumvent these issues and gain analytical insight, we consider an infinitely large population of simple cell-like reactors that do not grow or divide, each of which independently undergoes activation as described by Eq. (1). Based on experimental measurements on unprimed cultures (Fig. 3B), we introduce two additional parameters: $p_{0}$, which accounts for the initial proportion of activated cells, and $\tau_{+}$, which accounts for the initial waiting period. At t=0, each reactor has a probability $p_{0}$ to be T6SS activated, and an inactive reactor waits for $\tau_{+}$ before commencing stochastic switching. The activated percentage of the population P(t) at time t≥0 is


(2) $$P(t)=\left\{ \begin{array}{cc} p_{0} & \text{if}\;t<\tau_{+},\\ p_{0}+(1-p_{0})\left(1-e^{-(t-\tau_{+})\lambda_{+}}\right) & \text{if}\;t\geq \tau_{+}. \end{array}\right.$$

For both ES401 and FQ-A002, the measurements of activated population percentage at 0.5 h and 1 h in Fig. 3B are averaged to obtain their respective $p_{0}$ values, and all measurements are used in least-square regressions to estimate their respective $\lambda_{+}$ and $\tau_{+}$ (all values reported in Fig. 3 caption).

#### Structure assembly and deployment stage

The second stage in the T6SS biochemical model describes T6SS structure assembly and deployment. Previous studies in T6SS${}^{+}$ strains of *Pseudomonas aeruginosa*, *V. cholerae*, and *Acinetobacter baylyi* have shown that the effects of T6SS structural proteins on T6SS assemblies can be categorized in two ways: (1) the number of T6SS assemblies is driven by the abundance of proteins, such as the spike protein VgrG and effector proteins, which are present in low copy numbers, (2) the length of the sheath in a functional structure mainly depends on the abundance of multimeric proteins such as Hcp and VipAB (29, 30, 44, 58). To incorporate these observations in our model, we attribute new T6SS assemblies to the appearance of a low abundance structural protein, which we assume is synthesized at a constant rate, $\lambda_{s}$. We further assume that being in the activated state, all other component proteins are sufficiently abundant and thus do not limit the assembly. In terms of deployment, we consider that each T6SS structure can be independently fired at a constant rate, $\lambda_{f}$. Since each functional T6SS structure has a sheath apparatus, the term sheath is used interchangeably with the T6SS structure. Thus, we have the following stochastic processes for the number of sheaths *N*,


(3) $$\begin{array}{rl} & N\overset{\lambda_{s}}{⟶}N+1,\\ & N\overset{\lambda_{f}N}{⟶}N-1 \end{array}$$

In a large population of cell-like reactors undergoing both stages of the T6SS reactions (Eqs. (1)–(3)), the probability mass density function of the number of sheaths per cell tends toward a steady state at the long time limit. The rate of approaching steady state is determined by $\lambda_{+}$ and $\lambda_{f}$ (SI Appendix Section 3 and 4). The model predicts the average sheath number increases over time in a T6SS${}^{+}$ bacteria population with low initial T6SS activation. The steady-state average number of sheaths is ${\bar{N}}_{\infty }=\lambda_{s}/\lambda_{f}$. We cannot determine $\lambda_{s}$ or $\lambda_{f}$ separately in our experiments. Instead, we estimate ${\bar{N}}_{\infty }$ from the sheath averages at 6 h for both strains (Fig. 3C). To parametrize the model in the simplest and the most general manner, we choose ${\bar{N}}_{\infty }$ to be the same for both strains: ${\bar{N}}_{\infty }=3.5$. Note that Eq. (1) focuses only on the activation process, and Eqs. (3) neglect T6SS degradation processes independent of deployment. However, T6SS deactivation and degradation could be added in future work if required.

### The ABM with internal T6SS model

T6SS activity at the subcellular level directly affects the ability of T6SS${}^{+}$ populations to kill and thus influences the spatial structures on the length scale of the microbial colony (17, 26, 39, 40, 56). To investigate the multi-scale interplay between subcellular T6SS dynamics, cellular growth, and intercellular interactions, we integrate the T6SS biochemical model into an ABM (for more details, see SI Appendix Section 3 and 4). We choose our in-house ABM primarily for the ease of developing and testing the internal dynamics of the cells. While our ABM is restricted to 2D, it suffices for our present purpose of applying the internal model of T6SS to monolayer colonies. In addition to imposing T6SS-dependent interaction rules among dueling cells similar to those established in existing ABMs (17, 26, 56), each cell in our ABM undergoes internal stochastic reactions (Eqs. (1) and (3)) governing the assembly and deployment of the T6SS arsenal (Fig. 1B). We represent each cell as a spherocylinder (a cylinder with hemispherical ends) growing in a monolayer on a viscous substrate. Cell growth is modeled as elongation along the cylinder axis according to the adder model (68), while the radius of the cell is kept constant. As the cells grow and come into contact with one another, the mechanical interactions cause them to move and rotate and potentially restrict their growth (SI Appendix Fig. S4). Cell growth is also constrained by the carrying capacity of the 2D environment.

Each cell maintains the internal state variables G and N, T6SS activation state and sheath number, respectively, and carries out internal stochastic reactions (Eqs. (1) and (3)) at each time step in the simulation. If a cell fires a sheath, the target is randomly selected among the neighboring cells in contact. It is also possible for the cell to miss the neighbors and fire into the intercellular milieu. If the target is a clonemate, it survives; if the target is a nonclonal competitor cell, it ceases cellular function but participates in the mechanistic interactions for a time $\tau_{\mathrm{lys}}$ until it completes lysis and disintegrates. At division, the daughter cells inherit the mother cell’s T6SS activation state *G*, and the mother cell randomly distributes its sheaths with equal probability to the two daughters. The internal T6SS reactions are also coupled to a cell’s physiology via growth. In maintaining a T6SS arsenal, we assume that the energetic cost of T6SS protein expression and assembly is dominant over the cost of firing structures and maintaining the T6SS genes in the genome. A T6SS active cell has a penalized growth rate $r_{0}^{\prime }$ that decreases linearly with T6SS production rate $\lambda_{s}$, i.e. $r_{0}^{\prime }=r_{0}-c\lambda_{s},$ where $r_{0}$ is the base growth rate of the strain if it did not produce T6SS and c is the cost coefficient.

### Model predictions and comparison with experimental data

#### Slow activation rate limits T6SS effectiveness

Results in the previous sections indicate that the relatively slower surface activation rate in FQ-A002 is an important factor that led to the observation that wildtype FQ-A002 is outcompeted by wildtype ES401 under the unprimed condition (Fig. 2A–D). To test whether our model captures this experimental observation, we simulate competitions between ES401 and FQ-A002 and between their *vasA* mutants under unprimed and primed conditions, as in the experiments presented in Fig. 2A–D. We create different computational strains to represent ES401, FQ-A002, and their *vasA*${}^{-}$ counterparts, based on previously estimated parameters with several adjustments. T6SS parameters for simulated wildtype ES401 are $\left(p_{0},\lambda_{+},\lambda_{s},\lambda_{f},\tau_{\mathrm{lys}})=(10\%,0.6\;h^{-1},21\;h^{-1},6\;h^{-1},0.5\;h\right)$, and parameters for simulated wildtype FQ-A002 are $\left(p_{0},\lambda_{+},\lambda_{s},\lambda_{f},\tau_{\mathrm{lys}})=(5\%,0.25\;h^{-1},21\;h^{-1},6\;h^{-1},0.5\;h\right)$. To simulate a *vasA*${}^{-}$ strain, we set $\lambda_{f}=0$ in the corresponding wildtype strain so that the mutant cells cannot attack using T6SS but still pay a growth penalty for expressing T6SS proteins. More details on ABM parametrization in **Materials and methods** and SI Appendix Section 4.

Using a square periodic domain, we simulate the interior of the colony in a wildtype ES401 vs. FQ-A002 coincubation in both unprimed and primed conditions for an equivalence of 24 h. These simulations exhibit spatial characteristics qualitatively similar to both unprimed and primed biological assays (Fig. 2A–B). Averaging 100 simulations under unprimed conditions, ES401 occupies 91.4% ± 1.8% of the total area, compared with 99.3% in the experiment. In primed simulations, ES401 occupies 53.2% ± 4.5% of the total area, compared with 46.6% in the experiment. Simulated coincubation of ES401 *vasA*${}^{-}$ vs. FQ-A002 *vasA*${}^{-}$ also captures the well-mixed spatial structure as observed in the corresponding biological assays (Fig. 2C).

**Fig. 4.**
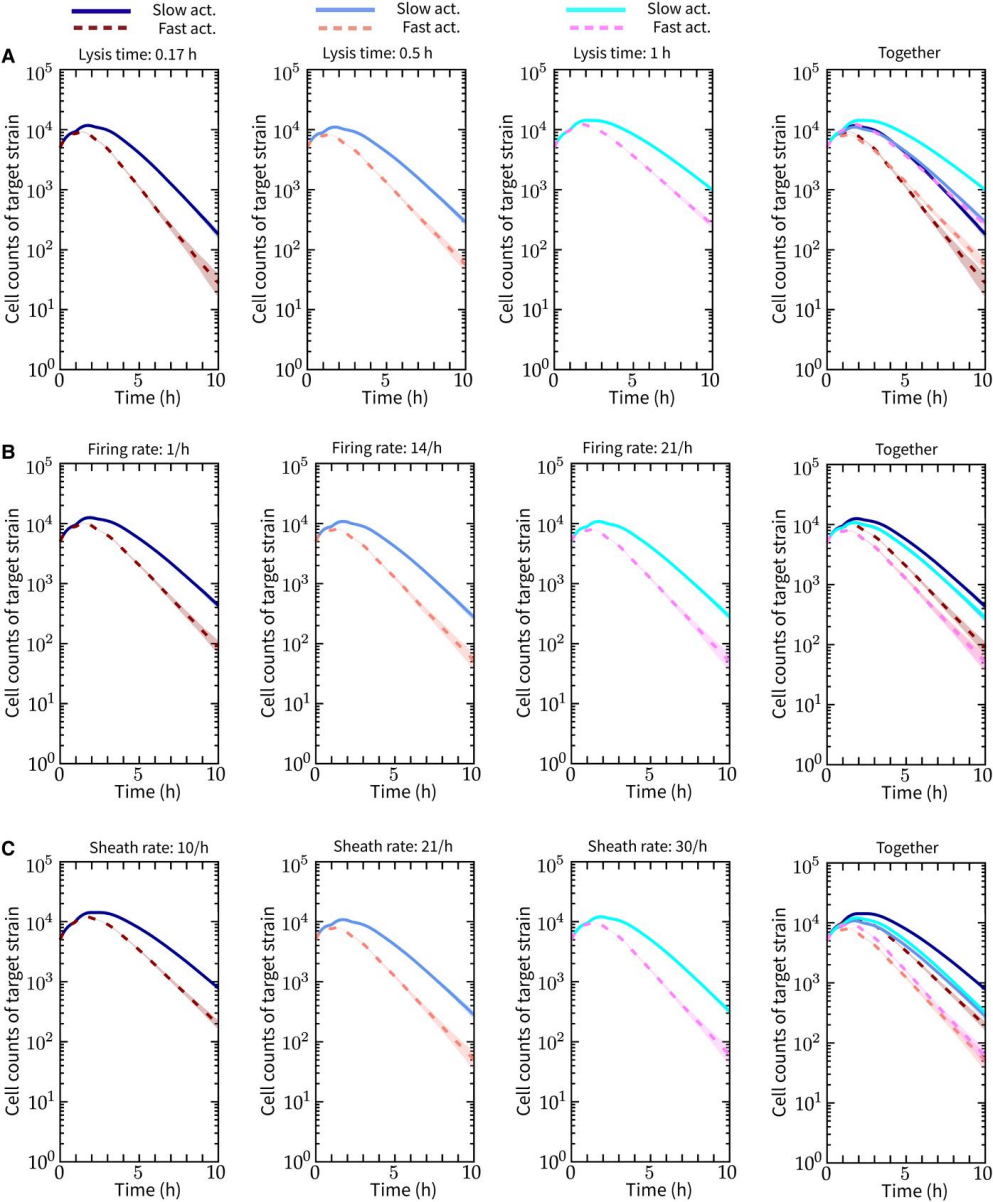
The effect of activation rate on lethal vs. target competition is consistent when other T6SS parameters are perturbed. A series of lethal vs. target coincubation experiments is simulated under the unprimed condition in confined spaces. In each scenario, two types of coincubation are simulated and the target strain populations are compared: one coincubation has a faster activating lethal strain ($\lambda_{+}=0.6\;h^{-1}$), and the other has a slower activating lethal strain ($\lambda_{+}=0.25\;h^{-1}$). Only the target population curves are shown. All curves are averaged over 50 independent simulations. In all the subfigures, the last panel on the right shows the combined data of the previous three. A) While keeping $\lambda_{s}=21\;h^{-1},\lambda_{f}=7\;h^{-1}$, lysis time is varied $\tau_{\mathrm{lys}}=0.17,0.5,1\;h$ across the first three panels, from left to right. B) While keeping $\lambda_{s}=21\;h^{-1},\tau_{\mathrm{lys}}=0.5\;h$, firing rate is varied $\lambda_{f}=1,14,21\;h^{-1}$ across the first three panels, from left to right. C) While keeping $\tau_{\mathrm{lys}}=0.5\;h$, sheath rate is varied $\lambda_{s}=10,21,30\;h^{-1}$, across the first three panels, from left to right. We set $\lambda_{f}=\lambda_{s}$ in each scenario. For other simulation parameters, see SI Appendix Tables S1 and S3.

We observed that the slower surface activation leads to FQ-A002 being less effective than ES401 in eliminating nonlethal targets. To show this in the ABM, we simulate wildtype vs. *vasA*${}^{-}$ pairs under unprimed conditions, similar to the experiments in Fig. 2E. As shown in Fig. 4, the *vasA*${}^{-}$ strain coincubated with a slower activating lethal strain grows to a higher peak population, maintains a higher population, and survives for a longer time, compared with that grown with a faster activating lethal strain. At later times, as the lethal population reaches full activation and the contact among cells is fully established, the *vasA*${}^{-}$ strains in both types of coincubation exhibit similar declining trends in population. To test if the effect of activation speed persists when other T6SS parameters are perturbed, we perform additional simulations by keeping the respective activation rates for both slower and faster-activating strains, and varying parameters $\lambda_{s}$ and $\lambda_{f}$ in the lethal strain and $\tau_{\mathrm{lys}}$ in the target strain.

Regardless of the parameter combination, when the activation rate is varied (individual panels in Fig. 4), we observed a persistent trend that the slower activating lethal strain allows the target strain to grow to a higher population level. We also noticed additional advantages of activating faster for the lethal strain. Evident in the last panel in Fig. 4A with all curves plotted together, a faster activating lethal strain is able to suppress the target population with lysis time of 1 h as effectively as a slower activating lethal populating competing against a target population with much shorter lysis times (0.17 h, 0.5 h). When the sheath rate is varied, we also found that having a faster activation rate and a relatively small sheath rate can achieve the same effect in target suppression as having a high sheath rate but a slower activation rate (Fig. 4B). The effect of a faster activation rate in enhancing target suppression can be similarly observed when the firing rate is varied (Fig. 4B). Taken together, these findings suggest that, for initially inactive, mutually lethal competitors, having a faster activation rate is a superior competitive strategy compared with having a slower activation rate, regardless of variations in the lysis time or the speed of sheath production and firing. In addition, having a faster activation can enhance the ability of the lethal strain to eliminate the target population even when its sheath production or firing rates are low or when the target strain’s lysis time is long.

#### Spatial environment of competition affects target survival

**Fig. 5.**
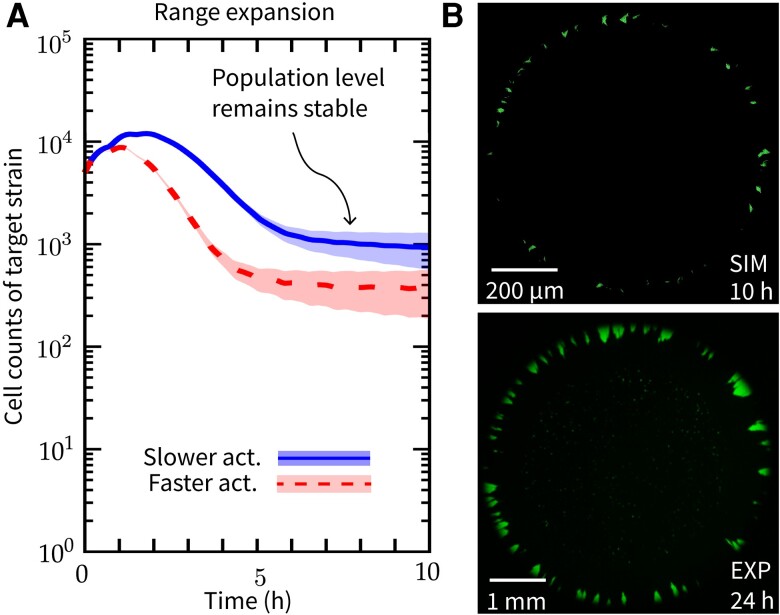
Target strains survive longer when competing against slow-activating lethal strains in a range expansion. A) The cell counts of a target strain over time when it competes against two lethal strains in a range expansion; all results are averaged over 50 simulations. Colors correspond to the activation rate of the lethal strain in the coincubation: blue = lethal strain activates slower with $\lambda_{+}=0.25\;h^{-1}$, red = lethal strain activates faster with $\lambda_{+}=0.6\;h^{-1}$. The shaded region on each curve shows ±1 SD. B) Top: a representative simulation image of the slow activating ($\lambda_{+}=0.25\;h^{-1}$) lethal (hidden) vs. target (shown) coincubation in range expansion. Scale bar is 200 µm. Bottom: a fluorescence microscopy image of the ES114 target strain following a coincubation with the ES401 inhibitor strain; scale bar is 1 mm. Unprimed strains were mixed at a 1:1 ratio and coincubated for 24 h on LBS agar plates. For more details and simulation parameters, see SI Appendix Section 2, movie S3, and Tables S1 and S3.

Bacteria compete in many different arenas, including environments where cells have space to expand their range as competition occurs and those where competition is limited to a confined space, e.g. host colonization sites. In simulating wildtype vs. *vasA*${}^{-}$ competitions, we observe that the spatial environment plays a significant role in determining the survival of the target strain. In a confined spatial geometry, the wildtype strain eventually eliminates the target population (Fig. 4). However, if the coincubation is allowed to grow in a range expansion, we find that a small population of target cells remains even after several hours of coincubation (Fig. 5A). Even though the lethal cells can eliminate all target cells in the interior of the colony, a small number of target cells can survive at the edge of the coincubation spot because only target cells at the boundary of these microcolonies come into contact with lethal cells, allowing the target cells bordered by clonemates to grow into the open territory and reproduce. (Fig. 5B, top).

The survival of target cells at the edge of a colony has also been consistently observed in the laboratory assays of lethal vs. target coincubation (Fig. 5B, bottom), albeit at different sizes and time scales from the simulations. We show that the edge survival phenomenon can be consistently observed by performing a series of simulations of increasing system size. However, as we expected, the numbers and sizes of the target strain microcolonies, and their temporal dynamics, vary depending on the initial conditions and likely other simulation parameters (SI Appendix Section 2, Fig. S3). Importantly, these findings demonstrate the utility of our ABM to replicate competitive outcomes under different spatial restrictions that are ecologically relevant.

### Cost determines the competitive fitness of T6SS production strategies

Variations in growth rates among bacterial strains can have significant impacts on competitive outcomes. While natural variations are common across bacterial strains, in this section, we focus on isolating the effect of a growth penalty due to T6SS activity. We consider intraspecific competitions among two fully activated lethal strains with identical cost coefficient c and base growth rate $r_{0}$ but different T6SS production rate $\lambda_{s}$. We may expect a tradeoff between the cost of T6SS production and its benefit in eliminating competitors.

We perform a parameter sweep using the ABM to understand the competitive fitness landscape in the context of a T6SS growth cost. We simulate a lethal resident strain with a fixed T6SS production rate of $\lambda_{s,\mathrm{res}}=20\;h^{-1}$, and a competitor strain with $\lambda_{s,\mathrm{comp}}$ varying in the range $\left[0,\lambda_{s,\mathrm{res}}\right]$. To measure the lethality of the competitor strain, we use a dimensionless parameter $\beta =\lambda_{s,\mathrm{comp}}/\left(\lambda_{s,\mathrm{res}}+\lambda_{s,\mathrm{comp}}\right)$. For the range of $\lambda_{s,\mathrm{comp}}$ that we consider, β∈[0,0.5]. We also rescale the cost coefficient as $\hat{c}=c/c_{\max}\in [0,1]$, where $c_{\max}=r_{0}/\lambda_{s,\mathrm{res}}$. To configure the competitor strain, we uniformly sample the parameter space of $\left\{\beta ,\hat{c}\;|0\leq \beta \leq 0.5,0\leq \hat{c}\leq 1\right\}$. We assess the competitive outcome by $\phi =(N_{\mathrm{res}}-N_{\mathrm{comp}})/(N_{\mathrm{res}}+N_{\mathrm{comp}})$, where $N_{\mathrm{res}}$ and $N_{\mathrm{comp}}$ are final cell counts of the resident and competitor strain, respectively. ϕ=1,0,−1 indicate resident strain dominance, coexistence, and competitor strain dominance, respectively.

**Fig. 6.**
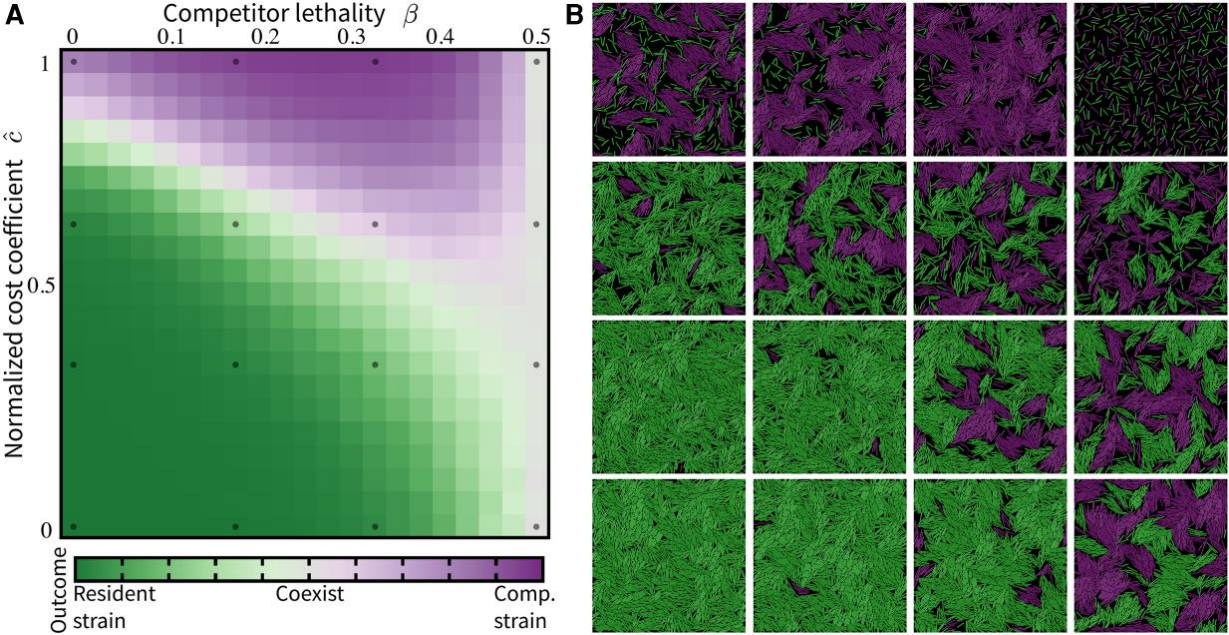
Competitive outcomes and surviving mechanisms are affected by the cost of T6SS production. A) Phase space of competitive outcome in mutually lethal competitions as a function of two dimensionless parameters, $\hat{c}$ and *β*, where $\hat{c}$ is the normalized cost coefficient and *β* characterizes the lethality of the competitor strain. All simulations are run for an equivalence of 10 h. Final cell counts $N_{\mathrm{res}}$ and $N_{\mathrm{comp}}$, for the resident and competitor strain, respectively, are collected and averaged over 50 independent trials for each parameter combination $\left\{\beta ,\hat{c}\right\}$. The competitive outcome is determined by $\phi =(N_{\mathrm{res}}-N_{\mathrm{comp}})/(N_{\mathrm{res}}+N_{\mathrm{comp}})$, with ϕ=1,0,−1 indicating resident strain dominance, coexistence, and competitor strain dominance, respectively. B) Representative simulation images from across the parameter space. For simulation parameters, see SI Appendix Tables S1 and S4.

In Fig. 6A, we show competitive outcome *ϕ* as a function of *β* and $\hat{c}$. In region along the diagonal of parameter space of $\left\{\beta ,\hat{c}\right\}$, the two competing strains can have different $\lambda_{s}$ values but still coexist because they strike a similar balance in the growth vs. T6SS production tradeoff. Above this diagonal region, the competitor strain (low producer) dominates, and below this diagonal, the resident strain (high producer) dominates. When β≈0.5, both strains coexist at any value of $\hat{c}$ due to comparable T6SS production levels.

Comparing simulations from across the parameter space (Fig. 6B), we find various mechanisms through which one strain can dominate or coexistence can be achieved. When the cost of producing T6SS is low, the resident strain dominates by having a higher T6SS production, suppressing the faster-growing competitor that maintains a smaller T6SS arsenal (Fig. 6B, lower left). With cost being low, when two strains coexist, cells grow and interact in T6SS-dependent manners to form spatially separated microcolonies, similar to what we observed in the primed coincubation of wildtype ES401 vs. wildtype FQ-A002 (Figs. 2B; 6B, center to lower right). When the cost is high, the competitor strain dominates by outgrowing the resident strain, which pays a heavy price for producing many T6SS structures (Fig. 6B, top left to center). With the high cost, cell growth can become extremely slow in high producers; thus, two competition strains coexist because the initial population barely grows to establish intercellular contacts (Fig. 6B, top right).

## Discussion

Built upon in vitro experimental data, the biochemical model contains the essential variables in subcellular T6SS dynamics: (1) the speed of T6SS activation and (2) the speed at which structures are built and fired during competition. Experimentally, we show that *V. fischeri* exhibits strain-specific variations in the speed of T6SS activation upon entering a viscous environment and in the average number of T6SS structures cells harbor. We hypothesized that the difference in activation speed plays a dominant role in strain competition and survival: the faster activator (ES401) has a competitive advantage by taking the first shots. Using in vitro and in silico experimental data, we provide evidence to corroborate this hypothesis (Figs. 2–5). When populations are fully activated at the start of the competition, competing factors such as the speed at which T6SS structures being generated and fired, and the cost of T6SS production, balance against one another in determining the competition outcome (Fig. 6).

Although the engineering of lab mutants has become standard research practice, there are yet limitations in practical feasibility and control when probing a biological system. To systematically investigate the effects on competitions by those T6SS-related mentioned above, we integrate the biochemical model into an ABM and use it to generate and test hypotheses about T6SS activity that would be difficult to address experimentally. We systematically vary T6SS-related parameters in the ABM to study the tradeoff between the biological cost of T6SS structure production and its competitive benefits (Fig. 6). Although there have been estimates of the energetic cost of firing T6SS (69), the growth penalty of expressing T6SS remains understudied and largely unknown (70). However, the phase diagram in Fig. 6 could be used as a tool either to predict the competitive outcome of two lethal strains if the growth cost of T6SS can be quantified or to predict the growth cost based on observed coincubation results. The growth rate is only one potential cost of utilizing T6SS (29, 70). Our model allows for the incorporation of different forms of cost, such as arrested cell cycles due to uptake of DNA of lysed prey cells (29), and DNA damage in T6SS expressing cells in the presence of environmental stressors (70).

Our ABM, combined with the internal T6SS model, can be a flexible investigative tool. By incorporating diffusion and intercellular communication, the ABM can consider other T6SS activation mechanisms such as iron-dependent gene expression, temperature fluctuations, and quorum sensing (34). It can also be modified to generate T6SS-related behaviors such as the tit-for-tat strategy (24, 56), reuse of secreted proteins in sister cells (30), feedback control of T6SS assembly via sensing the intracellular level of Hcp (71), and contact-independent toxicity (20). In addition to probing the biochemical parameters of T6SS, our model can be adapted to explore how bacteria may survive an encounter with a T6SS${}^{+}$ competitor. Although several reports have described observations of protection against T6SS attacks (19, 72–76), defensive mechanisms have yet to be parameterized in ABMs. Our model can be used to investigate T6SS resistance by incorporating factors that lead to the emergence of diverse protective capabilities, including those derived from heritable mechanisms, e.g. point mutations, gene amplification, and horizontal gene transfer, as well as physiological differences within a clonal population that are stochastic or influenced by hysteresis (77–79).

The ABM also enables us to investigate variables in competition not related to T6SS, such as changes in the geometry of the competition arena. The differences in target survival when competition occurs in a confined space and when it is in a range expansion (Fig. 5) provide some insight into competitions occurring within a host. The simulation results suggest that if multiple strains enter a confined host colonization site, the target population may be quickly eliminated because it cannot avoid contact with the lethal strain. These results underscore the importance of considering the geometry of the competitive environment, which substantially impacts the outcomes in addition to T6SS dynamics.

As a concluding remark, we have designed the T6SS biochemical model to be general and economical so that it can be adapted and applied to T6SSs in other microorganisms, serving as the foundation for more complex T6SS biochemistry to be investigated. Combined with the ABM, our model can easily incorporate new facets of T6SS-related mechanisms as they are discovered in this active area of research.

## Materials and methods

All data reported in this study can be found in the supplementary information and dataset. The simulation codes are freely available online at github.com/ylunalin/BacSim-T6SS.

### Media and growth conditions

*Vibrio fischeri* strains were grown in LBS medium at 24 °C, and antibiotics were added to media for *V. fischeri* selection as described previously (80). For selection in *V. fischeri* cultures, chloramphenicol, kanamycin, and erythromycin were added to LBS medium at final concentrations of 2 µg ml^−1^, 100 µg ml^−1^, and 5 µg ml^−1^, respectively.

### Coincubation assays

*Vibrio fischeri* strains containing the indicated plasmid or chromosomal markers were grown overnight on LBS agar plates supplemented with the appropriate antibiotic at 24 °C. For each biological replicate, overnight cultures were started from a single colony and grown overnight in LBS supplemented with the appropriate antibiotic. Before the start of coincubation assays, cultures were either subcultured once more into liquid LBS for 6 h (unprimed treatments) or spotted onto an LBS agar plate for 6 h (primed treatments) as indicated. For each coincubation, strains were normalized to an OD${}_{600}$ of 1.0, mixed at a 1:1 ratio, and 5 µl of the mixture was spotted on LBS agar plates and incubated at 24 °C. At the indicated time points, coincubation spots were either imaged using fluorescence microscopy, or total CFU counts were quantified using serial dilutions spotted onto media selective for the desired strain.

### Fluorescence microscopy

Fluorescence microscopy images of coincubation spots were imaged with a trinocular zoom stereo microscope equipped with a Nightsea fluorescence adapter kit for green and red fluorescence detection. Images were taken using an OMAX 14MP camera with OMAX ToupView camera control software. Single-cell images of VipA_2-GFP sheaths were taken either on an upright Olympus BX51 microscope outfitted with a Hamamatsu C8484-03G01 camera and a 100X/1.3 Oil Ph3 objective lens with cells prepped on a standard 1 mm glass slide (Fig. 3A), or on an inverted Nikon Ti2 microscope outfitted with a Hamamatsu ORCA Fusion sCMOS camera and a CFI plan apo lambda 100X oil objective lens with cells prepped on a 35 mm glass-bottomed dish (Fig. 3B) (81). Brightness and contrast adjustments were made uniformly across all images in a given experiment, and color changes were made by adjusting the LUT value to either “green” for ES401 or “magenta” for FQ-A002 in FIJI.

### Parametrization of simulated ES401 and FQ-A002 in the ABM

We use $\tau_{+}=1\;h$ for both simulated ES401 and FQ-A002 strains because the activated percentage in either biological ES401 or FQ-A002 population starts to increase after 1 h. The difference in $\tau_{+}$ estimates for the two strains is below our experimental time resolution (Fig. 3B). The rate of surface activation in a biological strain depends on the experimental conditions. The particular experiments to quantify this parameter used a sealed Petri dish for imaging (Fig. 3B), which likely limited the oxygen supply to the bacteria. As the experimental setups in Fig. 2 and Fig. 3A,C are different from this, we increase the estimated activation rates of both ES401 and FQ-A002 (Fig. 3) by five-fold while maintaining the ratio between the two. We base the value of $\lambda_{s}\approx 21\;h^{-1}$ in our computational strains on publicly available microscopy video data that visualize sheath assembly and firing in *V. cholerae* (23). The strain-specific firing rates are determined by $\lambda_{f}=\lambda_{s}/{\bar{N}}_{\infty }$. We use the estimate ${\bar{N}}_{\infty }=3.5$ for both ES401 and FQ-A002, the firing rates for the two strains are identically $\lambda_{f}=6\;h^{-1}$. For $\tau_{\mathrm{lys}}$, we use an intermediate value, 0.5 h, which is between the limiting values in a previous study that investigates the effect of lysis speed on T6SS effectiveness (17). Furthermore, we let $\lambda_{s}$ and $\tau_{\mathrm{lys}}$ be identical in both simulated ES401 and FQ-A002 strains because the two biological strains exhibit similar killing rates in coincubation with nonlethal targets (Fig. 2E). To simulate unprimed cells, after seeding the initial population at the start of the simulation, we randomly set a cell to be activated with probability $p_{0}$. We let the inactive cells undergo stochastic switching as described in Eq. (1). In contrast, we set every cell in the initial population to be activated at the start to simulate primed cells. The base growth rate $r_{0}$ is estimated from T6SS${}^{-}$*V. fischeri* strains and T6SS growth penalty c is adjusted so that the lethal strains’ penalized growth rate $r_{0}^{\prime }$ are similar to those of biological strains ES401 and FQ-A002 (8). The simulation domain in Fig. 2A–C is 388 µm × 388 µm and has periodic boundary conditions. However, we only use the central domain of 200 µm × 200 µm to compute the percentage of area occupied to avoid any edge effect. For a summary of ABM parameters, see SI Appendix Table S1. For specific parameters used in Figs. 2 and 6, see SI Appendix Tables S2 and S4, respectively. For specific parameters used in Figs. 4 and 5, see SI Appendix Table S3.

## Supplementary Material

pgad195_Supplementary_Data
