# Supplementary material for: A subcellular biochemical model for T6SS dynamics reveals winning competitive strategies
Source: PNAS Nexus. 2023 Jun 13;2(7):pgad195. doi: 10.1093/pnasnexus/pgad195 (PMC10335733; doi:10.1093/pnasnexus/pgad195)
Supplement: pgad195_Supplementary_Data [file pgad195_supplementary_data.zip › PNASNEXUS-PNASNEXUS-2022-00352RR-s01.pdf]

Supplementary Information for

A subcellular biochemical model for T6SS dynamics reveals winning competitive strategies

Yuexia Luna Lin, Stephanie N. Smith, Eva Kanso, Alecia N. Septer, Chris H. Rycroft

Corresponding Authors:
Chris H. Rycroft
E-mail: chr@math.wisc.edu
Alecia N. Septer
E-mail:alecia_septer@med.unc.edu

This PDF file includes:

Figs. S1 to S5
Tables S1 to S4
Legends for Movies S1 to S3
Legend for Dataset S1
SI References

Other supplementary materials for this manuscript include the following:

Movies S1 to S3
Dataset S1

Part I: Supplemental data and further discussions

1. Additional experimental and simulation data for Fig. 2 in the main text

We include the experimental data presented in this manuscript in the following dataset:
SI Dataset S1 (Lin-Smith-et_al_experimental_data)

A. Competition outcomes vary due to strain-specific variations in T6SS surface activation response. In the main text, we introduce two treatments of inocula to prepare them for competition assays (Fig. 2 in the main text). In the unprimed treatment, inocula are raised in liquid culture, mixed, and spotted directly onto the agar surface for competitive coincubation. In contrast, inocula can also be primed before mixing and competing by a clonal incubation period on an agar surface. Priming on a viscous surface activates and enhances type VI secretion system (T6SS) structure formation and T6SS-dependent killing in *V. fischeri* (1–3). We also perform competition assays between $vasA^-$ strains under either treatment. Since *vasA* gene disruption blocks the expression of a T6SS structural component, mutants with this disruption cannot make functional T6SS even though they still express the majority of the T6SS-associated proteins. These assays serve as controls, showing that factors besides T6SS-dependent interactions do not give rise to the spatial organization and population dynamics observed in wildtype vs. wildtype competitions under the corresponding conditions. Whether the coincubation is under primed or unprimed conditions, competition outcomes between $vasA^-$ strains of ES401 and FQ-A002 are similar.

In Fig. 2 in the main text, we report the results of *vasA* mutants of ES401 and FQ-A002 competing under the unprimed treatment. In Fig. S1A, we show an example microscopy image of such an assay under primed treatment. We also show four additional examples of agent-based model (ABM) simulation of unprimed wildtype vs. wildtype competition (Fig. S1B), primed wildtype vs. wildtype competition (Fig. S1C), and $vasA^-$ vs. $vasA^-$ competitions (Fig. S1D). In $vasA^-$ vs. $vasA^-$ ABM simulations, T6SS is not engaged in inter-cellular interactions. Thus, we do not distinguish between primed or unprimed conditions because both scenarios' algorithmic details are identical. Finally, we include two simulation movies to show the temporal dynamics of competition assays. The simulation parameters can be found in Tables S1 and S2.

Movie S1. Unprimed wildtype ES401 vs. wildtype FQ-A002 competition.

Movie S2. Primed wildtype ES401 vs. wildtype FQ-A002 competition.

B. Lethal strains ES401 and FQ-A002 have similar growth rates in lethal vs. target coincubations. In the main text, we describe experiments where we coincubated lethal vs. target pairs of (1) wildtype ES401 vs. FQ-A002 $vasA^-$, and (2) wildtype FQ-A002 vs. ES401 $vasA^-$ (Fig. 2E in the main text). There, we observed that ES401 $vasA^-$ was able to maintain its population level during 1 h–2 h, whereas the CFU counts of FQ-A002 $vasA^-$ continued to decline during the same time window. This is consistent with our hypothesis that strain-specific variations in T6SS surface activation response affect the population dynamics of the interacting bacterial strains. In particular, this result supports our claim that FQ-A002, having a slower surface activation response, allows its targets to survive longer and in larger numbers when compared to the faster-activating ES401. To show that different growth rates are the reason for the observed difference in target decline, we present data of the colony forming units (CFU) of the lethal strains in each coincubation in Fig. S2, showing that the growth rates of the two lethal strains are similar in these coincubation assays.

2. Spatial environment of competition affects target survival

In the main text, we show that nonlethal targets can survive T6SS attacks from lethal competitors in a range expansion, where target cells are protected by clonemates and can grow into territories free of lethal cells. In addition to the snapshot we provide in Fig. 5 in the main text, we show a series of range expansion coincubation between a lethal strain and a target strain. In Fig. S3, we vary the initial spot size from small to large and show that regardless of the initial system size, at some point during the coincubation, the target strain in the bulk of the colony will be eliminated, leaving some small microcolonies surviving at the edge. However, the number, the sizes, and the temporal dynamics of these edge microcolonies depend on the initial spot size and simulation duration. These characteristics of the surviving target microcolonies also depend on factors such as cell density, growth rate, and T6SS-related parameters.

For the smallest configuration (initial spot size 97 µm), we perform additional simulations to complete a total of 105 independent realizations of duration T=24 h. We find that the target strain survival probability, i.e., the probability of having a non-zero target strain population at the end of the simulation period, is 88.6%. Inspecting the simulation results, we find that when target cells survive, they *always* survive in microcolonies at the outer edge of a colony. We include an example simulation movie that demonstrates this phenomenon of target cells surviving at the colony edges. Simulation parameters that are not specified here can be found in Table S1 and S3.

Movie S3. Nonlethal target cells survive T6SS attacks at the edges of a range expansion.

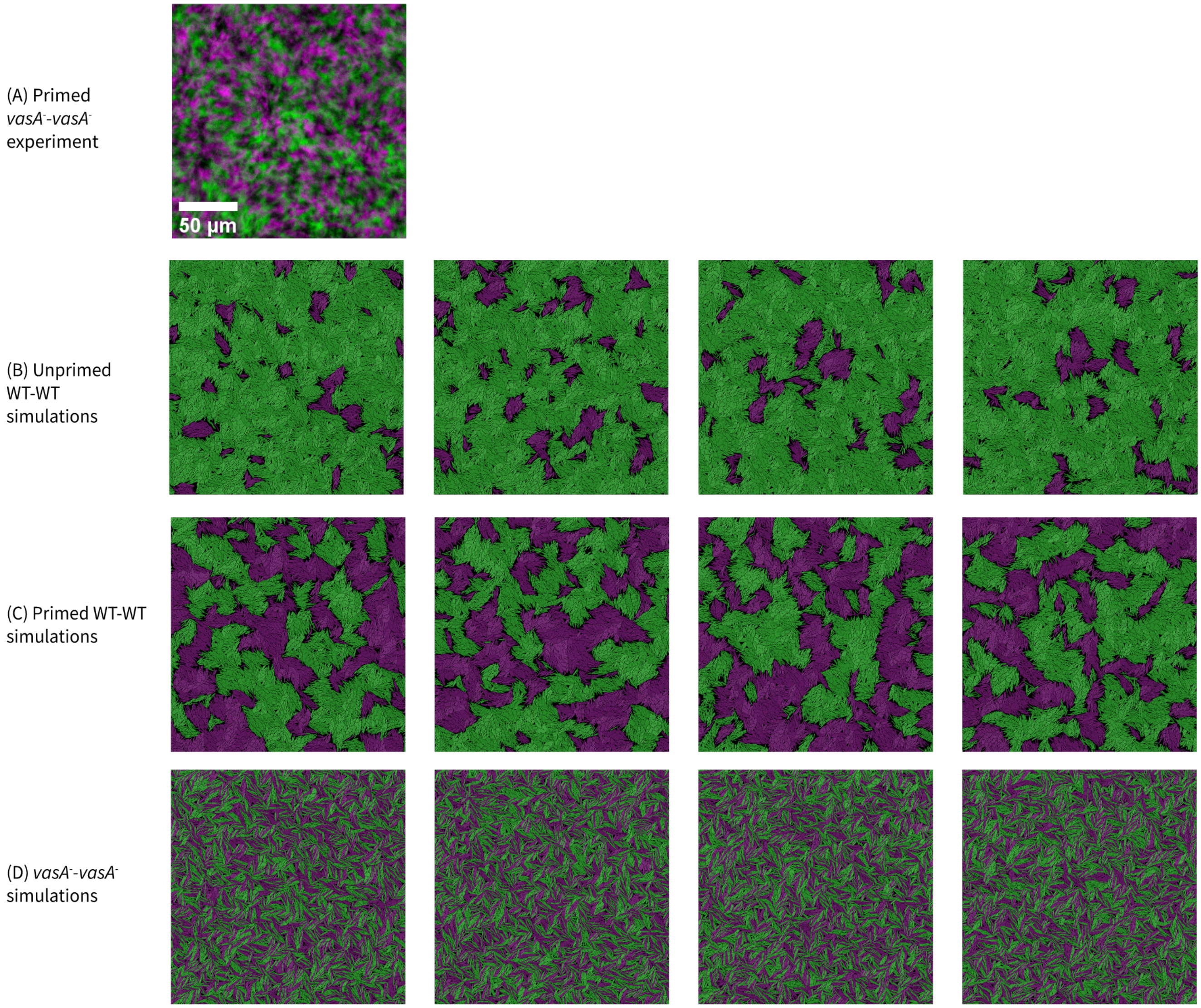

Fig. S1. Additional experimental and simulation data on wildtype vs. wildtype, *vasA*$^-$ vs. *vasA*$^-$ coincubations under unprimed and primed conditions. (A) Example microscopy image of primed *vasA*$^-$ vs. *vasA*$^-$ competition, which serves as a control assay in the experiments described in Fig. 2A–D in the main text. Images of four independent simulations at $T = 24\,\mathrm{h}$ of unprimed wildtype vs. wildtype simulations (B), primed wildtype vs. wildtype simulations(C), and *vasA*$^-$ vs. *vasA*$^-$ competitions (D). (B)–(D) share the same scale bar as (A).

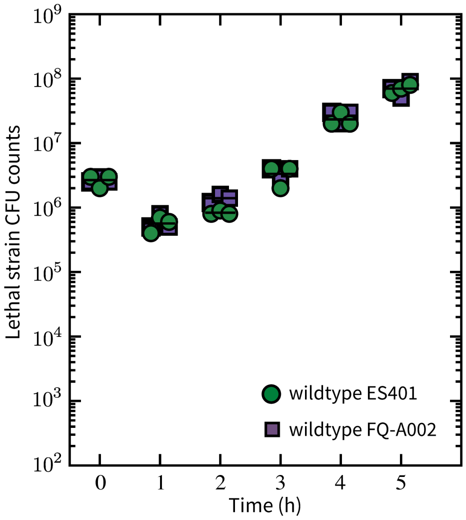

Fig. S2. Lethal wildtype strains ES401 (green) and FQ-A002 (purple) CFU counts show similar growth rates in lethal vs. target coincubation. Wildtype ES401 (green circles) is coincubated with FQ-A002 *vasA*$^-$. Wildtype FQ-A002 (Purple squares) is coincubated with ES401 *vasA*$^-$. Both pairs are grown under the unprimed condition. CFU counts of the wildtype strains are shown. Other experimental protocols are reported in the caption for Fig. 2E and the Materials and Methods section in the main text.

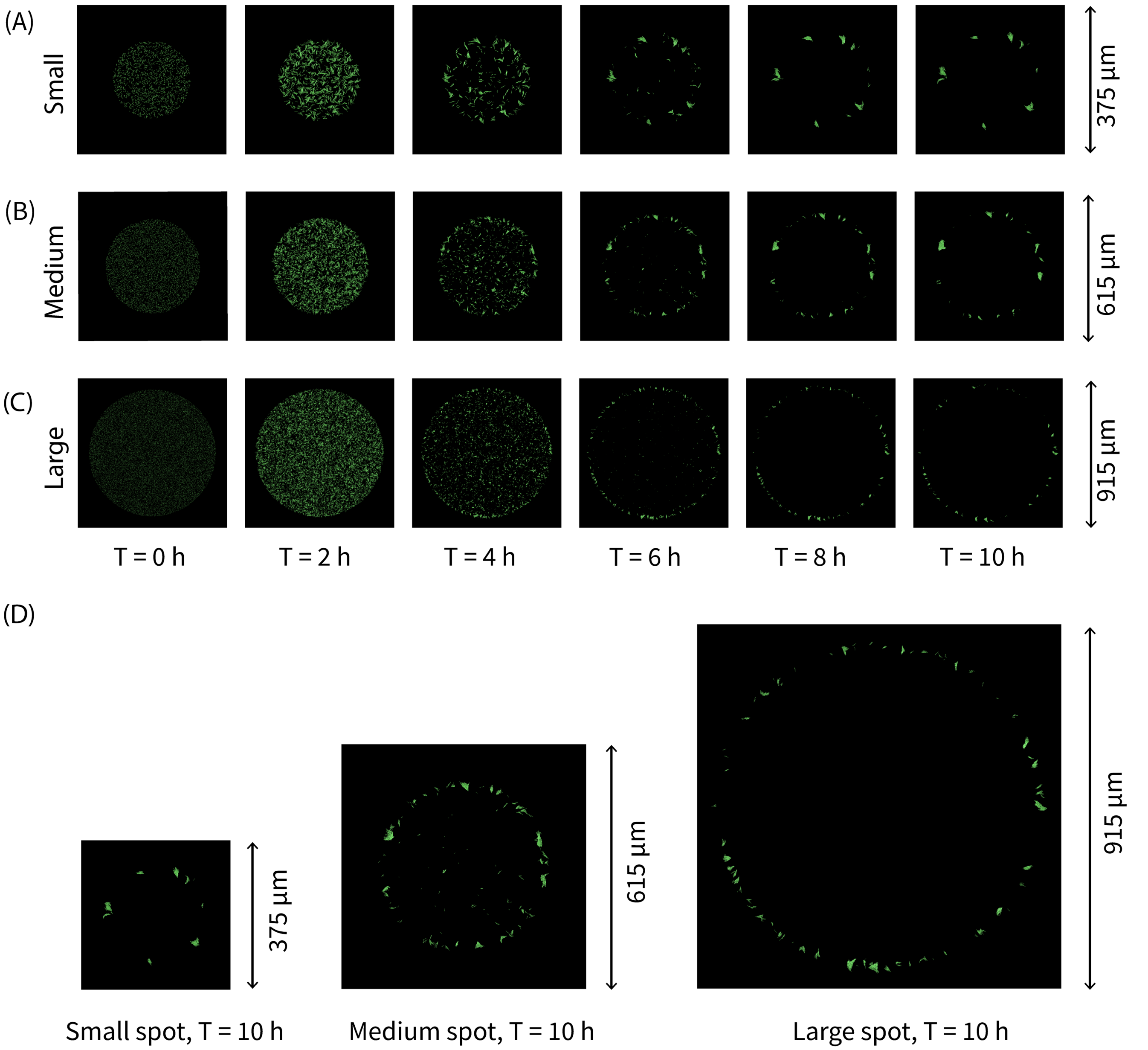

Fig. S3. Edge survival is consistently observed in T6SS$^+$ lethal (not visible) vs. nonlethal target (green) competition simulations with varying system sizes. The initial spot sizes increase from panel (A) to panel (C). (A) The initial spot has a radius of $97\,\mu\mathrm{m}$ and contains $1250{:}1250$ lethal vs. target cells. (B) The initial spot has a radius of $194\,\mu\mathrm{m}$ and $5000{:}5000$ lethal vs. target cells. (C) The initial spot has a radius of $388\,\mu\mathrm{m}$ and $20000{:}20000$ lethal vs. target cells. Snapshots at $T{=}0\,\mathrm{h}, 2\,\mathrm{h}, 4\,\mathrm{h}, 6\,\mathrm{h}, 8\,\mathrm{h}, 10\,\mathrm{h}$ are visualized for each system size. Note that initial cell number density is maintained across all simulations. (D) The three last snapshots at $T{=}10\,\mathrm{h}$ are rescaled to reflect their relative sizes. Besides initial spot size, other simulation parameters are identical across the three scenarios (see Table S1 and Table S3).

Part II: Details of the subcellular T6SS biochemical model and the ABM

3. Subcellular T6SS biochemical model

Consider the two-stage T6SS biochemical model,

$$\begin{aligned} G^- &\xrightarrow{\lambda_+} G^+, \\ N &\xrightarrow{\lambda_s} N{+}1, \\ N &\xrightarrow{\lambda_f N} N{-}1, \end{aligned} \qquad [1]$$

where N is the number of T6SS structures (sheaths), and the binary state variable G represents the cells being in T6SS active (G^+) or inactive (G^-) state. T6SS structures are produced at a constant rate λ_s and fired at a rate proportional to the number of structures, $\lambda_f N$. Consider a cell in the activated state G^+, in a small time interval $(t, t+\delta)$, the probability of producing a sheath is $\lambda_s(\delta + o(\delta))$. The probability of a sheath being fired is $N\lambda_f(\delta + o(\delta))$. The probability of not producing any sheath is $1 - \lambda_s(\delta + o(\delta))$, and that of not using any sheath is $1 - N\lambda_f(\delta + o(\delta))$. Therefore, the probability of neither producing nor using any structure is

$$(1 - \lambda_s(\delta + o(\delta)))(1 - N\lambda_f(\delta + o(\delta))) \approx 1 - \delta(\lambda_s + N\lambda_f) \qquad [2]$$

where we have assumed the probability of having two or more reactions within δ is small, i.e., $\lim_{\delta\to 0} \frac{o(\delta)}{\delta} = 0$. Denote the probability of having $N = n$ structures at time t as $P_n(t)$. Then

$$\begin{aligned} P_n(t+\delta) &= P_{n-1}(t)(\lambda_s(\delta + o(\delta))) + P_{n+1}(t)((n+1)\lambda_f(\delta + o(\delta))) \\ &\quad + P_n(t)(1 - \lambda_s(\delta + o(\delta)) - n\lambda_f(\delta + o(\delta))) \\ &\approx \lambda_s\delta P_{n-1}(t) + (n+1)\lambda_f\delta P_{n+1}(t) + P_n(t)(1 - \lambda_s\delta - n\lambda_f\delta). \end{aligned} \qquad [3]$$

We approximate time derivative of $P_n(t)$ by taking the limit of $\delta \to 0$,

$$\begin{aligned} \frac{\partial P_n(t)}{\partial t} &= \lim_{\delta\to 0} \frac{P_n(t+\delta) - P_n(t)}{\delta} \\ &= -(\lambda_s + n\lambda_f)P_n(t) + \lambda_s P_{n-1}(t) + (n+1)\lambda_f P_{n+1}(t). \end{aligned} \qquad [4]$$

This is the master equation satisfied by the probability mass function of the number of sheaths for $n \geq 0$. Next, we will derive the governing equation for the probability generating function of this probability distribution and solve for the generating functions for $\lambda_f = 0$ and $\lambda_f > 0$. The generating function for a probability distribution is

$$G(s,t) = \sum_{n=-\infty}^{\infty} s^n P_n(t). \qquad [5]$$

We will use the following expressions in our derivations:

$$\sum_{n=-\infty}^{\infty} s^n n P_n(t) = s\frac{\partial G(s,t)}{\partial s}, \qquad [6]$$

$$G(1,t) = \sum P_n(t) = 1, \qquad [7]$$

$$\langle n \rangle = \left.\frac{\partial G(s,t)}{\partial s}\right|_{s=1} \equiv G'(1,t), \qquad [8]$$

$$\langle n(n-1) \rangle = \left.\frac{\partial^2 G(s,t)}{\partial s^2}\right|_{s=1} \equiv G''(1,t), \qquad [9]$$

$$\langle n^2 \rangle = \left.\frac{\partial}{\partial s}\left(s\frac{\partial G(s,t)}{\partial s}\right)\right|_{s=1}. \qquad [10]$$

To proceed, we make use of a step operator, which is a linear operator commonly used in solving master equations such as Eq. [4]. Details of the techniques for handling master equations, e.g., the generating function and the step operator, can be found in standard textbooks for stochastic processes such as Ref. (4). The linear step operator $E^l[\cdot]$ where $l \in \mathbb{Z}$, the set of integers, can be applied to a function $f(n)$, where the support n can run over the real numbers. The linear operator can be defined as

$$\begin{aligned} E^l[f(n)] &= f(n+l), \\ (E^l - 1)[f(n)] &= f(n). \end{aligned} \qquad [11]$$

Step operations have the property

$$\sum_{n=-\infty}^{\infty} s^n (E^k - 1)[f(n)] = (s^{-k} - 1) \sum_{n=-\infty}^{\infty} s^n f(n). \qquad [12]$$

Rewriting the time derivative of the probability distribution in Eq. [4] with the linear step operator we introduced in Eqs. [11], we have

$$\frac{\partial P_n(t)}{\partial t} = (E-1)[n\lambda_f P_n(t)] + (E^{-1}-1)[\lambda_s P_n(t)]. \tag{13}$$

Taking the partial time derivative of Eq. [5] and using Eq. [13], we have

$$\begin{aligned}\frac{\partial G(s,t)}{\partial t} &= \sum_{n=-\infty}^{\infty} s^n \left\{(E-1)[n\lambda_f P_n(t)] + (E^{-1}-1)[\lambda_s P_n(t)]\right\} \\ &= (s^{-1}-1)\sum_{n=-\infty}^{\infty} s^n(n\lambda_f P_n(t)) + (s-1)\sum_{n=-\infty}^{\infty} s^n(\lambda_s P_n(t)) \\ &= \lambda_f(1-s)\frac{\partial G(s,t)}{\partial s} + \lambda_s(s-1)G(s,t).\end{aligned} \tag{14}$$

In the second line, we have used the property of jump functions, Eq. [12], and in the third line, we have used Eq. [6] for the first term, and the definition of $G(s,t)$ in Eq. [5] for the second term. The last equation above, Eq. [14], is a first-order partial differential equation (PDE) for $G(s,t)$, which can be solved by the method of characteristics, with an appropriate initial condition

$$G(s,0) = G_0(s). \tag{15}$$

For $\lambda_f = 0$, Eq. [14] reduces to an ordinary differential equation. Solving the initial value problem with the initial condition in Eq. [15] yields

$$G(s,t) = e^{\lambda_s(s-1)t}G_0(s). \tag{16}$$

Differentiating $G(s,t)$ with respect to s and evaluating the derivatives appropriately (Eqs. [8] & [10]), we find that the mean and the variance of the number of T6SS structures are

$$\langle N(t)\rangle = \left.\frac{\partial G(s,t)}{\partial s}\right|_{s=1} = \lambda_s t + \langle N(0)\rangle, \tag{17}$$

$$\sigma_N^2(t) = \left.\frac{\partial}{\partial s}\left(s\frac{\partial G(s,t)}{\partial s}\right)\right|_{s=1} - \langle N(t)\rangle^2 = \lambda_s t + \sigma_N^2(0), \tag{18}$$

respectively, where $N(0)\rangle = G_0'(1)$ and

$$\sigma_N^2(0) = \langle N(0)^2\rangle - \langle N(0)\rangle^2 = G_0''(1) + G_0'(1) - (G_0'(1))^2. \tag{19}$$

In the expression of initial variance, $\sigma_N^2(0)$, we have used identities in Eqs. [8] & [9]. For $\lambda_f > 0$, Eq. [14] has a solution

$$G(s,t) = \exp\left\{\tfrac{\lambda_s}{\lambda_f}(s-1)(1-e^{-\lambda_f t})\right\} G_0((s-1)e^{-\lambda_f t}+1). \tag{20}$$

As we will see next, this is the generating function for a Poisson distribution. Differentiating Eq. [20] shows that the mean is

$$\langle N(t)\rangle = \left.\frac{\partial G(s,t)}{\partial s}\right|_{s=1} = \tfrac{\lambda_s}{\lambda_f}(1-e^{-\lambda_f t}) + e^{-\lambda_f t}\langle N(0)\rangle \tag{21}$$

and the variance is

$$\begin{aligned}\sigma_N^2(t) &= \left.\frac{\partial}{\partial s}\left(s\frac{\partial G(s,t)}{\partial s}\right)\right|_{s=1} - \langle N(t)\rangle^2 \\ &= \left(\tfrac{\lambda_s}{\lambda_f} + e^{-\lambda_f t}\langle N(0)\rangle\right)(1-e^{-\lambda_f t}) + e^{-2\lambda_f t}\sigma_N^2(0).\end{aligned} \tag{22}$$

Taken together, Eqs. [20], [21] & [22] indicate that in a system with $\lambda_f > 0$, the effect of the initial condition is damped out, or forgotten, exponentially at a rate of λ_f. The probability mass distribution of sheaths is a Poisson distribution with a time-dependent mean. In the long time limit, the steady state distribution has a mean $\overline{N}_\infty = \lambda_s/\lambda_f$, and a variance of the same value.

Next we consider the effect of cell division on the sheath number distribution. Assume a mother cell distributes each of its sheaths with equal probability to either of the daughter cells. The probability of a daughter cell receiving k out of the n sheaths in the mother cell is

$$\mathbb{P}_d(k|n) = \binom{n}{k}\frac{1}{2^n}. \tag{23}$$

The total probability of a newborn cell having k sheaths needs to account for the probability of the mother cell having n sheaths,

$$\mathbb{P}_d(k) = \sum_{n=0}^{\infty} \mathbb{P}_d(k|n)\mathbb{P}_m(n) \quad [24]$$

$$= \sum_{n=0}^{\infty} \frac{n!}{k!(n-k)!} \frac{1}{2^n} \frac{\overline{N}_{\infty}^n e^{-\overline{N}_{\infty}}}{n!} \quad [25]$$

$$= \frac{1}{k!} \left(\frac{-\overline{N}_{\infty}}{2}\right)^k e^{-\overline{N}_{\infty}} \sum_{n=0}^{\infty} \frac{1}{(n-k)!} \left(\frac{-\overline{N}_{\infty}}{2}\right)^{n-k} \quad [26]$$

$$= \frac{1}{k!} \left(\frac{\overline{N}_{\infty}}{2}\right)^k e^{-\overline{N}_{\infty}/2} \quad [27]$$

where $\overline{N}_{\infty} = \lambda_s/\lambda_f$ is the steady-state mean of the Poisson distribution in Eq. [21]. The subscripts d and m denote daughter and mother. Here we have assumed that the probability mass function of sheaths in mother cells has reached a steady state. We assume that the firing time scale is much faster than the cell cycle time so that the exponentially decaying terms in Eqs. [21] and [22] can be treated as zero. However, division disrupts the equilibrium in sheath distribution. The calculation above shows that the sheath number distribution of a synchronous population of cells immediately after division is also a Poisson distribution, with half of the original mean. Following the discussion above, this post-division distribution equilibrates to the steady state Poisson distribution with mean and variance $\overline{N}_{\infty}$ exponentially fast, with a convergence rate dependent on firing rate λ_f.

4. Description of the in-house agent-based model

In this section, we introduce our in-house ABM and discuss the integration of the T6SS biochemical model (Eqs. [1]). Our ABM is designed for microbial growth on two-dimensional (2D) surfaces such as agar plates, and it takes into account cell growth, division, and cell-cell and cell-substrate interactions. Under fast growth conditions, i.e. cell growth is unrestricted, the model of cell growth and division implemented in the ABM reduces to the so-called adder model (5). However, in surface growth, factors such as pressure induced by crowding, nutrient and space depletion, and waste accumulation can limit cell growth, increasing doubling time or stopping growth altogether. Later in this section, we will discuss the mechanism we use to constrain cell growth in our ABM.

A. Single cell ideal growth. We represent a cell as a spherocylinder with two hemispherical caps of radius R, and a body length l_{cyl}. The total cell length is $l = l_{\text{cyl}} + 2R$. We represent the growth of such a cell by elongation along its cylindrical axis and use l to represent cell size in lieu of cell volume. In a population of cells with an average growth rate r_0, the average doubling time is $\tau_c = \ln(2)r_0^{-1}$. Let l and l_b denote an individual cell's current length and length at birth, respectively. Cells are shown to grow exponentially, therefore, $l(t) = l_b e^{tr_0}$. Let l_0 be the population average cell length at birth, and l_d be the length at the time of division. A unified model of single-cell size regulation has been proposed by Ref. (5). The cell size at division is

$$l_d = 2^{1+\frac{\eta_t}{\tau_c}} l_b^{1-a} l_0^a, \quad [28]$$

where η_t is a noise in interdivisional time, and parameter a gives rise to several common size regulation models. For example, in the absence of noise, choosing $a = 1$ results in a model where cells divide at a critical length, i.e., $l_d = 2l_0$. This is called the sizer model because it results in tight control of birth sizes around average size l_0. Choosing $a = 0$ leads to constant growth time so that cells always double birth size, i.e., $l_d = 2l_b$. This is called the timer model because the growth time is tightly distributed around the mean. In the ABM, we choose $a = 0.5$, resulting in the so-called adder model. In this growth model, cells add a constant volume regardless of birth size. Therefore, cells that are larger than average at birth grow for a time shorter than average cell cycle, and vice versa. The adder model produces statistics that agree with experiments, in particular, coefficients of variance between size at birth and size at division and distributions of cell size and interdivisional time. For details, see the works of Amir and coworkers and references therein (5–8).

In simulating cell cycle using Eq. [28], we use a small Gaussian noise $\eta_t \sim \mathcal{N}(0, \sigma_t^2)$ to perturb the interdivisional time. The division is considered symmetric between the daughter cells. However, a small Gaussian noise in length $\eta_l \sim \mathcal{N}(0, \sigma_l^2)$ is used to perturb the daughter cell sizes at birth. To illustrate this, consider a mother cell of length l_d, symmetrically dividing into two daughters. Daughter 1 and daughter 2 have cell length at birth

$$l_{b,1} = \tfrac{1}{2} l_d + \eta_l, \qquad l_{b,2} = l_d - l_{b,1}, \quad [29]$$

respectively. After division, we perturb the orientations of daughter cells by a small Gaussian noise $\eta_\theta \sim \mathcal{N}(0, \sigma_\theta^2)$, to avoid artificial chaining. Values of the noise size parameters are summarized in Table S1.

B. Mechanical interactions and growth restriction in the ABM. Since we simulate cells growing on a two-dimensional surface, space availability is the most prominent factor limiting the growth. We develop two mechanisms to constrain individual cell growth based on factors related to space availability. First, we employ a pressure-based growth restriction factor to effectively stop cells from elongating when the space is depleted along its growth axis. To facilitate this discussion, we first describe mechanical interactions among cells, followed by the details of the growth restriction model, locally calculated for each cell based on the pressure it experiences. Later, we also introduce a carrying capacity-based growth restriction, which is applied as a global restriction to all the cells in a simulation.

B.1. Interactions within a population. As cells grow and divide, they experience mechanical forces from other cells as they come into contact and push on one another. Cells also experience damping forces due to contact with the viscous substrate. Let us denote cells in the ABM by $b_i, b_j, b_k, \ldots$ We assume that upon contact, cells undergo small deformations, and the material behaves as a linear elastic material with elastic modulus E, which has the dimension of force per area. When cells get pushed into each other either due to growth or due to mechanical interactions with neighboring cells, the inter-cellular contact between a pair of cells i and j causes a small deformation h in both cells. This can be detected in the simulation using positional and geometrical information about the cells. As shown in the schematic in Fig. S4A, the region enclosed by dashed curves indicates the total deformation needed to accommodate the pairwise contact. The maximum width H of this region, which can also be viewed as the overlap between two perfectly rigid spherocylinders, can be computed by standard contact detection methods. The deformation of each cell h is $h = \frac{1}{2}H$ since two contacting cells equally deform in the process. The strain in each cell can be obtained by scaling with a typical length $\frac{h}{R}$. Assuming that the contact area scales as $\sim R^{2*}$, the magnitude of the repulsive force exerted on cell i due to cell j scales as $|\mathbf{f}_{ij}| \sim ER^2\frac{h}{R} = ERh$. The direction along which the repulsive forces act in this pair of cells can be determined from the geometry (illustrated in Fig. S4A). We sum over all such forces on a cell and the viscous force it experiences to get the total force on this cell, $\mathbf{F}_i$.

The Newtonian dynamics of cell i are described by the equations of motion,

$$\begin{aligned} \dot{\mathbf{x}}_i &= \mathbf{v}_i, \\ m_i\dot{\mathbf{v}}_i &= \sum_{j\in J} \mathbf{f}_{ij} - \gamma_l \mathbf{v}_i, \end{aligned} \tag{30}$$

where $\mathbf{x}_i$ and $\mathbf{v}_i$ are the position and velocity of cell i, respectively. The cell mass is $m_i = \rho(\pi R^2 l_{i,\mathrm{cyl}} + \frac{4}{3}\pi R^3)$. γ_l is the viscous damping coefficient of the substrate, $\mathbf{f}_{ij}$ denotes the contact forces exerted by cell j on i, where j is in the set of cells that are in contact with i, denoted by J. The contact forces also induce torques in the cells. The angular dynamics is described by

$$\begin{aligned} \dot{\theta}_i &= \omega_i, \\ I_i\dot{\omega}_i &= \sum_{j\in J} \tau_{ij} - \gamma_r \omega, \end{aligned} \tag{31}$$

where θ_i and ω_i are the orientation and angular velocity of cell i, respectively. γ_r is the viscous damping coefficient for rotating cell bodies, I_i is the moment of inertia of a spherocylinder rotating about its center, perpendicular to the cylindrical axis,

$$I_i = \frac{2}{5}m_{i,\mathrm{sph}}R^2 + m_{i,\mathrm{sph}}(l_{i,\mathrm{cyl}}/2)^2 + \frac{1}{12}\left(3R^2 + l_{i,\mathrm{cyl}}^2\right)m_{i,\mathrm{cyl}}, \tag{32}$$

where $m_{i,\mathrm{sph}} = \frac{4}{3}\rho R^3$ is the sum of the masses of the two hemispherical caps, and $m_{i,\mathrm{cyl}} = \rho\pi R^2 l_{i,\mathrm{cyl}}$ is the mass of the cylinder. In the equation above, the first term is the moment of inertia of a sphere or that of two hemispheres. The second term applies the parallel axis theorem to translate the two hemispheres to either end of the cell. The third term is the moment of inertia of a cylinder. Using dimensional analysis (12), $\gamma_r \sim A\gamma_l$, where A has the dimension of area. Equations [30] & [31] make up the system of ODEs that describes the motion of a cell, and we solve these equations for each cell using an explicit forward Euler scheme.

B.2. Parameter estimation. The exact values of γ_l and γ_r are not important as long as the values provide sufficient damping in the system. We make use of an estimation procedure that provides reasonable values of these two parameters. In our mechanistic model above, we have the following parameters: growth rate r_0, average cell size l_0, cell radius R, cell density ρ, linear and rotational viscous damping coefficients γ_l, γ_r, and elastic modulus E. We can construct four dimensionless groups using Buckingham's Π theorem.

Using ρ, l_0, R, we can construct an average mass $m_0 = \rho(\pi R^2(l_0 - 2R) + \frac{4}{3}\pi R^3)$. Suppose a cell with mass m_0 is pushed by one end of an exponentially growing cell, and undergoes only translation (no rotation); the pushing force due to the growing neighboring cell $\sim m_0 r_0^2 l_0$ and the viscous damping force due to contact with the substrate $\sim \gamma_l r_0 l_0$. Taking the quotient of the two, we have a dimensionless parameter $p_1 = \gamma_l/(m_0 r_0)$. We set $p_1 = 100$, i.e. $\gamma_l = 100 m_0 r_0$ so that motions due to cell growth are sufficiently damped by viscous interactions. To set γ_r, we use the dimensionless group $p_2 = \gamma_r/(A_0\gamma_l) = 1$, where $A_0 = (l_0 - 2R)R + \pi R^2$ is the largest horizontal cross-section area of a cell of average cell size l_0. We assume that this is the typical area in contact with the viscous substrate.

*Our assumption of constant contact area leads to a Hookean contact model, a commonly-used model in discrete-element method simulations of particles (9). A more accurate description would assume that contact area depends on h, which can lead to, e.g., the Hertzian contact model (10). However, the Hertzian model is more computationally expensive to implement and has a limited effect on the dynamics of moving particle assemblies when compared to experimental results (11). Therefore, we make use of the simpler Hookean model.

Another dimensionless parameter, $p_3 = ER^2/(\gamma_l r_0 l_0)$, represents the ratio between repulsive elastic and damping forces. This parameter helps us determine the timestep restriction in the simulation. The maximum timestep should ensure the effects of elastic forces are properly resolved, leading to $dt_{\max} = m_0/(p_3\gamma_l)$. The last dimensionless parameter $p_4 = ER^2/(m_0 r_0^2 l_0)$ is the ratio between the elastic forces due to contact and pushing forces due to cell growth. Parameters p_3 and p_4 are important in addressing a common issue in ABMs of cell growth: cells may exhibit unrealistic overlaps after some time of growth. To alleviate this problem, we choose an elastic modulus E such that p_3 and p_4 are large enough, ensuring that cells are kept separate on the viscous substrate as they grow. We find that setting $p_3 = 10$ is sufficient to prevent significant overlaps among cells in our simulations, i.e. $E = 10\gamma_l r_0 l_0/R^2$, while still ensuring that the motions in the system are damped out by viscous interactions. We acknowledge that there is a large degree of freedom in choosing the values for these parameters. To check that these parameter values are fulfilling their regulating roles, before using our ABM for the study, we performed small tests to ensure resulting colony of cells does not exhibit nonphysical motions or overlaps with one another. Small perturbations in the values of these parameters may affect the actual numerical values output by the model but not the general trends we have observed from numerical experiments. The dimensionless parameters are summarized in Table S1, and values of dimensional parameters dt, γ_l, γ_r, and E, which depend on the morphology of the cells we simulate, are computed at the start of each simulation.

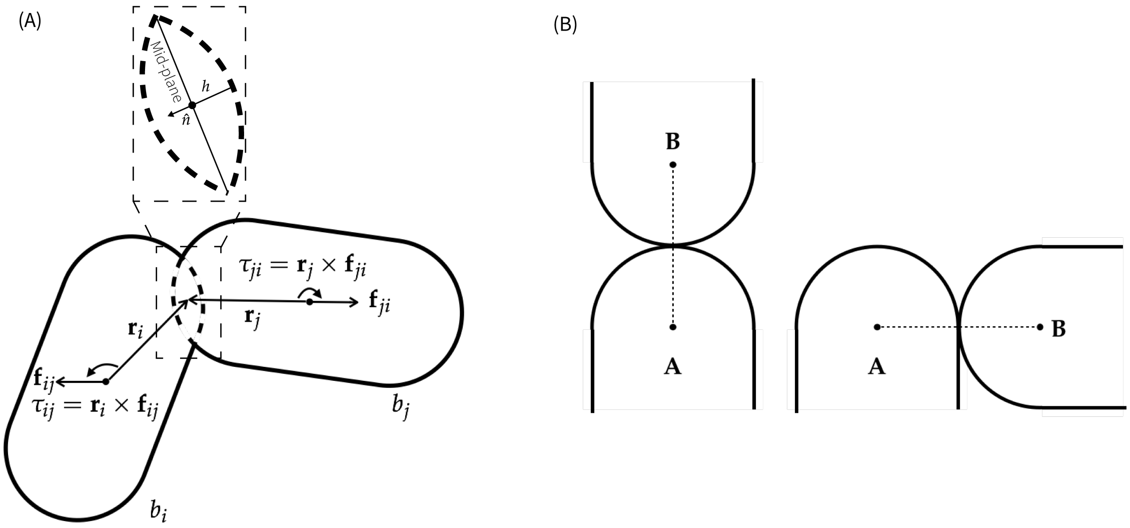

Fig. S4. (A) A schematic of cell-cell interaction. The amount of deformation needed to accommodate two cells in contact can be considered as the overlap between two spherocylinders (dashed region). The amount of deformation h of each cell is half of the maximum width of the overlapping region. $\hat{n}$ denotes the direction along which the repulsive forces are acting; it is perpendicular to the mid-plane of the overlapping region. Also indicated in the schematic are contact forces, moment arms, and resulting torques acting on each cell in contact. (B) Left: the contact force between cell A and cell B can potentially inhibit both cells because pressure due to contact is along the growth axis of both A and B. If the other end of a cell is also blocked, the pressure from growth will build up and stop the cell from elongating. Right: the contact force between A and B would only potentially inhibit the growth of cell B. However, the equal but opposite contact forces on cell A only apply a torque on the cell and do not inhibit its growth.

B.3. Mechanistic growth restriction. To further address the problem of unrealistic overlaps among cells in the late stages of growth, we find it is also necessary to impose a growth limit. Although restrictions in growth can be due to resource availability, waste accumulations, antibacterial chemicals, etc., the primary factor we focus on is the availability of space. Intercellular pressure can reach a high value in a crowded region of the bacterial colony, which can slow down and even stop growth altogether (13–15). The contact model described above provides a convenient way to impose a growth constraint based on local crowdedness. A similar mechanism to constrain growth has been used in other ABMs (16).

Here, we assume that inhibition of growth is due to mechanical forces exerted along the growth axis but independent of forces orthogonal to the axis (Fig. S4B). Let r denote the actual growth rate, $r = \beta_f r_0$, where $\beta_f \leq 1$ is a multiplicative growth restriction factor due to mechanical forces. Therefore, the actual interdivisional time is $\tau_c = (\log 2) r^{-1}$ (the small Gaussian perturbation not shown here). The expression for β_f is

$$\beta_f = \max\left(0, 1 - \frac{P_{\text{up}}\ P_{\text{down}}}{P_c(P_{\text{up}} + P_{\text{down}})}\right) \qquad [33]$$

where P_{up} and P_{down} are calculated from contact forces at a cell's upper and lower ends, respectively, and the critical pressure, P_c, determines when a cell stops growing. This form of growth restriction resembles the empirical model for growth restriction imposed by nutrient availability developed by Monod (17) and has the property that it requires pressure experienced by both ends of the cell to act together to stop growth. To give some physical intuition, if one end of the cell is blocked by neighbors, but the other end of the cell is free from contact with any cell, the cell should be able to grow toward the free side without any impediment. In fact, in this scenario, equation [33] evaluates to 1. Similar to the half-saturation constant in the Monod

nutrient restriction model (17), P_c can be considered a half inhibition constant—if the pressures experienced by both ends are P_c, then the cell growth rate is halved. The pressure along the growth (cylindrical) axis is computed by taking the projection of the contact force along the cell axis, and normalizing it by the cross-sectional area of the cell, πR^2. The critical pressure P_c is currently an unknown parameter, but it must be determined *a priori*. By empirical testing, we choose a small but sufficient value, $P_c = \xi_c E$ with $\xi_c = 5 \times 10^{-3}$, which helps prevent over-constraining cell growth. In the implementation of the ABM, we also add a fail-safe mechanism where if two cells become crossed at any point, we immediately set $\beta_f = 0$ for both cells.

B.4. Carrying capacity-based growth restriction. As indicated by Fig. S4 above, the force-based restriction model can arrest unrealistic growth and prevent cell overlaps when both ends of a cell are blocked by other cells. However, when the cell density is very high, for example, in a periodic domain after a long growth time, spherocylinder cells tend to align parallel to one another. In this scenario, the force-based model described above may not be effective since the pressure experienced by the cells is mainly on the lateral surfaces rather than the ends. When a simulation exhibits this degree of crowdedness, our model assumption of monolayer growth is most likely no longer valid. Our priority, then, is to arrest growth and prevent unrealistic cell overlaps. Instead of modifying the force-based mechanism, we impose an additional growth restriction factor based on the well-known concept of the carrying capacity of an ecological environment (18). We consider the total area of the simulation domain as the maximum carrying capacity A_0. Then, the penalty on the growth rate as this limit is approached is

$$\beta_a = 1 - \frac{A}{A_0}, \qquad [34]$$

where A is the total area occupied by all the cells, including those that are alive and those that are dead but not yet disintegrated. Similar to the calculations in the previous section, the actual growth rate r becomes

$$r = \beta_f \beta_a r_0. \qquad [35]$$

C. Integration of the T6SS biochemical model in the ABM. Here we expand on how we integrate the T6SS biochemical model into the internal state of cells in the ABM. The T6SS activation state G and apparatus number N are stored as internal state variables. As described in the main text, the activation probability is

$$P(t) = \begin{cases} p_0 & \text{if } t < \tau_0, \\ p_0 + (1 - p_0)\left(1 - e^{-(t-\tau_0)\lambda_+}\right) & \text{if } t \geq \tau_0, \end{cases} \qquad [36]$$

where the parameter p_0 represents the T6SS activity level in liquid culture and τ_0 takes into account a possible wait time before the activation process begins.

To incorporate these parameters in the simulation, each initial seed cell has a probability of p_0 to be active and if it is active, it initially possesses N_0 sheaths, where N_0 is a random number drawn from a Poisson distribution with a mean λ_s/λ_f. If there is a waiting period $\tau_0 > 0$ (which can be strain-specific or idiosyncratic to each cell), then during the waiting period $0 \leq t < \tau_0$, the cell does not engage in any biological processes. This means it does not grow, divide, activate T6SS assembly, form T6SS structures, or fire any structure. After the waiting period, during each timestep, each cell elongates its length according to

$$\frac{dl}{dt} = rl \qquad [37]$$

where r is the actual growth rate specific to each cell, subject to constraint due to space limitation and the cost of producing T6SS. We model the T6SS cost as a penalty on the base growth rate (16) so that a T6SS$^+$ cell that is active has a growth rate

$$r_0' = r_0 - c\lambda_s \qquad [38]$$

where c is the cost coefficient. Note that when a cell is in an inactive state, even if it can produce and fire T6SS structures, the base growth rate is unaltered since the T6SS penalty is due to expressing the T6SS. Combined with mechanical growth restriction, the cell-specific actual growth rate is

$$r = \beta_f \beta_a r_0'. \qquad [39]$$

In addition, during each timestep, the following events can occur with appropriate probabilities according to Eqs. [1]:

1. an inactive cell becomes activated,
2. an active cell produces a new T6SS apparatus,
3. a cell that has any T6SS apparatuses can fire one.

We require that a cell must be activated before it can produce sheaths, and a cell can only fire if it has one or more sheaths. Therefore, during a timestep dt, in inactivated cells, event 1 is the only possible event with probability $\lambda_+ dt$. In activated cells, event 2 occurs with probability $\lambda_s \beta_f \beta_a dt$, where the growth restriction factors β_f, β_a, due to crowding and depletion of space, respectively, have been multiplied to the base sheath rate λ_s. Event 3 is only possible in activated cells when the sheath number $N > 0$, with probability $\lambda_f N dt$. Events 2 and 3 can both occur at the same timestep.

Upon firing a T6SS apparatus, the attacking cell randomly selects one of its neighboring cells, i.e., those cells in contact, as the target. It can also fire into the environment without hitting any target. In our T6SS biochemical model, the average

number of sheaths per cell is regulated via a balance between production and firing. Translated to the ABM, we must simulate cells that can fire into the extracellular milieu even when isolated from neighboring cells. In doing so, we avoid an excessive accumulation of sheaths within an isolated cell, which leads to skewed sheath number distribution and increased firing frequency when the cell comes into contact with others later on.

To simulate this, we consider each neighbor cell and the extracellular milieu as a slot to fire into. A cell can fire a T6SS attack into each slot with equal probability. For example, if a cell has two neighboring cells, then the T6SS is fired with equal probability $\frac{1}{3}$ into either of the neighbors or the extracellular milieu. After each cell has its turn in the T6SS-dependent interaction, we survey the entire simulation and determine if each cell is still alive. If the target is a sister cell of the attacker, it remains alive. Otherwise, it is marked as dead. A dead cell ceases growth, division, and other internal processes immediately but still participates in the mechanical interactions in the simulation. An internal lysis timer starts counting from the moment a cell is marked as dead. The dead cell is removed from the simulation after lysis time τ_{lys} is reached, i.e. the cell has disintegrated.

Division occurs at the end of a timestep for any eligible cells. As mentioned before, during division, the sheaths of the mother cell are randomly assigned to the two daughter cells according to a binomial distribution, corresponding to giving each sheath to either daughter cell with equal probability.

D. Summary of ABM units and parameters. The simulating unit length can be matched to the physical length by rescaling with the average cell length of the bacteria. We estimate the average cell size of our experimental strains from microscopy images and report these values in Fig. S5. For simplicity, for all simulated strains, we use $3\,\mu\text{m}$ for the average cell length at birth and $0.6\,\mu\text{m}$ for the average width, which stays constant during growth. In the simulation, the bacteria are spherocylinders with constant radius $1\,\text{L}$. Thus the simulation unit length can be converted to a physical unit by $1\,\text{L} = 0.3\,\mu\text{m}$. We also let one unit simulation time be one hour in physical time, i.e. $1\,\text{T} = 1\,\text{h}$. Similarly, we can rescale the mass unit M in the ABM so that the simulation mass density ρ has a value of unity, $1\,\text{M}/\text{L}^3$.

Previous experiments (19) on which our current experimental methods are based estimated that the doubling time of a T6SS^- strain of *V. fischeri*, ES114, is approximately $39\,\text{min} \pm 5.8\,\text{min}$. The doubling time of the T6SS^+ strain FQ-A002 is estimated to be approximately $43\,\text{min} \pm 10\,\text{min}$ under identical experimental conditions (19). We adopt these growth rate estimates and use doubling time $39\,\text{min}$, i.e., growth rate $1.07\,\text{h}^{-1}$, as the base growth rate for all strains in the simulation. We further assume that the observed difference in doubling time can be attributed to diverting material and energy needed for growth to expressing T6SS-associated proteins, based on an estimate of $\lambda_s \approx 21\,\text{h}^{-1}$, we use Eq. [38] to estimate the default cost coefficient, $c \approx 0.005$.

We categorize and summarize all model parameters of the ABM in Table S1, and provide parameters used for the simulations in Figs. 2 & 4–6 in the main text in Tables S2, S3, & S4.

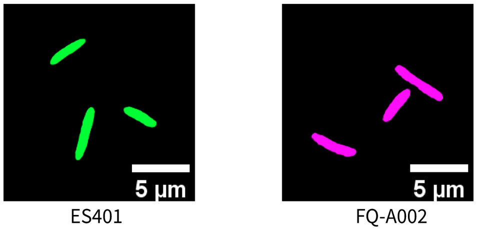

Fig. S5. Quantification of sizes of ES401 and FQ-A002 cells. Overnight cultures of wildtype ES401 and FQ-A002 harboring the GFP expressing plasmid pVSV102 were grown clonally in unprimed conditions prior to spotting $2\,\mu\text{L}$ onto an agarose pad. Cells were imaged in the FITC channel within $10\,\text{min}$ of spotting; length and width measurements were calculated using NIS Elements software. For strain ES401, 533 cells were analyzed across 10 individual images. ES401 $3.2\,\mu\text{m} \pm 1.1\,\mu\text{m}$, width $0.64\,\mu\text{m} \pm 0.12\,\mu\text{m}$ (left). For strain FQ-002, 512 cells were analyzed across 10 individual images. FQ-A002 length $3.5\,\mu\text{m} \pm 1.3\,\mu\text{m}$, width $0.65\,\mu\text{m} \pm 0.11\,\mu\text{m}$ (right). Representative images are shown.

Name	Symbol	Value	Unit
System parameters			
Domain size	L	Varying	μm
Boundary condition	-	Periodic or free	-
Simulation time	T	Varying	h
Initial cell number density	ρ_{num}	8.3 (default), varies	per $100\,\mu m^2$
Ratio between damping forces and the intercellular forces due to growth	p_1	100	dimensionless
Ratio between linear and rotational damping coefficients	p_2	1	dimensionless
Ratio between elastic forces and damping forces	p_3	10	dimensionless
Multiplier for critical pressure in growth rate regulation	ξ_c	5×10^{-3}	dimensionless
Noise size in doubling time perturbation	σ_t	0.02	h
Noise size in division length perturbation	σ_l	0.1	μm
Noise size in post-division orientation perturbation	σ_θ	0.01	rad
Cell mass density	ρ	1	$g\,cm^{-3}$
Cell physiology parameters			
Cell width	R	0.6	μm
Average cell length at birth	l_0	3.0	μm
Average base growth rate	r_0	1.07	h^{-1}
T6SS associated parameters			
Initial activation percentage (liquid culture activity level)	p_0	Varies	dimensionless
Activation rate	λ_+	Varies	h^{-1}
Structure synthesis rate	λ_s	Varies	h^{-1}
Structure firing rate	λ_f	Varies	h^{-1}
Structure synthesis cost coefficient	c	0.005 (default), Varies	dimensionless
Lysis time	τ_{lys}	0.5 (default), Varies	h

Table S1. A list of simulation parameters in the in-house ABM, with integrated subcellular T6SS biochemical model.

System parameters				
Name	Symbol	Value		Unit
Square domain size	L	388		μm
Boundary condition	-	Periodic		-
Simulation time	T	24		h
Initial cell number density	ρ_{num}	8.3		per $100\,\mu m^2$

T6SS associated parameters				
Name	Symbol	ES401	FQ-A002	Unit
Initial activation percentage	p_0	10%	5%	dimensionless
Activation rate	λ_+	0.6	0.25	h^{-1}
Structure synthesis rate	λ_s	21	21	h^{-1}
Structure firing rate	λ_f	6	6	h^{-1}
Structure synthesis cost coefficient	c	0.005		dimensionless
Lysis time	τ_{lys}	0.5		h

Table S2. Parameters used in the ABM simulations for Fig. 2 in the main text, and in Fig. S1B–D, for unprimed wildtype cells. To simulate primed cells, the initial activation percentage p_0 is adjusted to $p_0 = 100\%$. To simulate *vasA*$^-$ cells, firing rate λ_f is adjusted to $\lambda_f = 0$.

System parameters				
Name	Symbol	Range expansion	Confined spaces	Unit
Domain size	L	194 (initial spot radius)	388 (square domain size)	μm
Boundary condition	-	Free	Periodic	-
Simulation time	T	10	10	h
Initial cell number density	ρ_{num}	8.3	6.6	per $100\,\mu m^2$

T6SS associated parameters				
Name	Symbol	Lethal strain	Target strain	Unit
Initial activation percentage	p_0	0%	0%	dimensionless
Activation rate	λ_+	0.25 (slow), 0.6 (fast)	0	h^{-1}
Structure synthesis rate	λ_s	10, 21 (Fig. 5), 30	0	h^{-1}
Structure firing rate	λ_f	1, 7 (Fig. 5), 14, 21	0	h^{-1}
Structure synthesis cost coefficient	c	0.005	0	dimensionless
Lysis time	τ_{lys}	0.17 (Fig. 5), 0.5, 1	0	h

Table S3. Parameters used in the ABM simulations for Figs. 4 & 5 in the main text. The values of λ_s, λ_f, and τ_{lys} for Fig. 5 are indicated as such, the others are for Fig. 4. Values of all other parameters are the same for both figures.

System parameters				
Name	Symbol	Value		Unit
Square domain size	L	78		μm
Boundary condition	-	Periodic		-
Simulation time	T	10		h
Initial cell number density	ρ_{num}	8.3		per $100\,\mu m^2$

T6SS associated parameters				
Name	Symbol	Resident strain	Competitor strain	Unit
Initial activation percentage	p_0	100%		dimensionless
Structure synthesis rate	λ_s	20	$[0, 20]$	h^{-1}
Structure firing rate	λ_f	20	$[0, 20]$	h^{-1}
Structure synthesis cost coefficient	c	$[0, 0.0533]$		dimensionless
Lysis time	τ_{lys}	0.5		h

Table S4. Parameters used in the ABM simulations for Fig. 6 in the main text.

References

1. L Speare, S Smith, F Salvato, M Kleiner, AN Septer, Environmental viscosity modulates interbacterial killing during habitat transition. *mBio* **11**, e03060–19 (2020).
2. L Speare, et al., Host-like conditions are required for T6SS-mediated competition among *Vibrio fischeri* light organ symbionts. *mSphere* **6**, e01288–20 (2021).
3. S Smith, F Salvato, A Garikipati, M Kleiner, AN Septer, Activation of the type VI secretion system in the squid symbiont *Vibrio fischeri* requires the transcriptional regulator TasR and the structural proteins TssM and TssA. *J. Bacteriol.* **203**, e00399–21 (2021).
4. T Raúl, Introduction to master equations in *Stochastic Numerical Methods*, eds. P Colet, T Raúl. (John Wiley & Sons, Ltd), pp. 235–260 (2014).
5. A Amir, Cell size regulation in bacteria. *Phys. Rev. Lett.* **112**, 208102 (2014).
6. PY Ho, A Amir, Simultaneous regulation of cell size and chromosome replication in bacteria. *Front. Microbiol.* **6** (2015).
7. F Barber, PY Ho, AW Murray, A Amir, Details matter: Noise and model structure set the relationship between cell size and cell cycle timing. *Front. Cell Dev. Biol.* **5**, 92 (2017).
8. PY Ho, J Lin, A Amir, Modeling cell size regulation: From single-cell-level statistics to molecular mechanisms and population-level effects. *Annu. Rev. Biophys.* **47**, 251–271 (2018).
9. LE Silbert, et al., Granular flow down an inclined plane: Bagnold scaling and rheology. *Phys. Rev. E* **64**, 051302 (2001).
10. KL Johnson, *Contact Mechanics.* (Cambridge University Press), (1985).
11. CH Rycroft, AV Orpe, A Kudrolli, Physical test of a particle simulation model in a sheared granular system. *Phys. Rev. E* **80**, 031305 (2009).
12. GI Barenblatt, *Scaling.* (Cambridge University Press), (2003).
13. BI Shraiman, Mechanical feedback as a possible regulator of tissue growth. *Proc. Natl. Acad. Sci.* **102**, 3318–3323 (2005).
14. D Volfson, S Cookson, J Hasty, LS Tsimring, Biomechanical ordering of dense cell populations. *Proc. Natl. Acad. Sci.* **105**, 15346–15351 (2008).
15. D Yang, AD Jennings, E Borrego, ST Retterer, J Männik, Analysis of factors limiting bacterial growth in PDMS mother machine devices. *Front. Microbiol.* **9**, 871 (2018).
16. WPJ Smith, et al., The evolution of the type VI secretion system as a disintegration weapon. *PLOS Biol.* **18**, e3000720 (2020).
17. J Monod, The growth of bacterial cultures. *Annu. Rev. Microbiol.* **3**, 371–394 (1949).
18. NF Sayre, The genesis, history, and limits of carrying capacity. *Annals Assoc. Am. Geogr.* **98**, 120–134 (2008).
19. L Speare, et al., Bacterial symbionts use a type VI secretion system to eliminate competitors in their natural host. *Proc. Natl. Acad. Sci.* **115**, E8528–E8537 (2018).
